# Evolution of Plant P-Type ATPases

**DOI:** 10.3389/fpls.2012.00031

**Published:** 2012-02-21

**Authors:** Christian N. S. Pedersen, Kristian B. Axelsen, Jeffrey F. Harper, Michael G. Palmgren

**Affiliations:** ^1^Center for Membrane Pumps in Cells and Disease – PUMPKIN, Danish National Research Foundation, Aarhus UniversityAarhus, Denmark; ^2^Bioinformatics Research Centre (BiRC), Faculty of Science and Technology, Aarhus UniversityAarhus, Denmark; ^3^Department of Plant Biology and Biotechnology, Faculty of Life Sciences, Center for Membrane Pumps in Cells and Disease – PUMPKIN, Danish National Research Foundation, University of CopenhagenFrederiksberg C, Denmark; ^4^Swiss-Prot Group, Swiss Institute of BioinformaticsGeneva, Switzerland; ^5^Department of Biochemistry and Molecular Biology, University of NevadaReno, NV, USA

**Keywords:** evolution, P-type ATPases, plants, salt tolerance, Na^+^ pumps

## Abstract

Five organisms having completely sequenced genomes and belonging to all major branches of green plants (Viridiplantae) were analyzed with respect to their content of P-type ATPases encoding genes. These were the chlorophytes *Ostreococcus tauri* and *Chlamydomonas reinhardtii*, and the streptophytes *Physcomitrella patens* (a non-vascular moss), *Selaginella moellendorffii* (a primitive vascular plant), and *Arabidopsis thaliana* (a model flowering plant). Each organism contained sequences for all five subfamilies of P-type ATPases. Whereas Na^+^ and H^+^ pumps seem to mutually exclude each other in flowering plants and animals, they co-exist in chlorophytes, which show representatives for two kinds of Na^+^ pumps (P2C and P2D ATPases) as well as a primitive H^+^-ATPase. Both Na^+^ and H^+^ pumps also co-exist in the moss *P. patens*, which has a P2D Na^+^-ATPase. In contrast to the primitive H^+^-ATPases in chlorophytes and *P. patens*, the H^+^-ATPases from vascular plants all have a large C-terminal regulatory domain as well as a conserved Arg in transmembrane segment 5 that is predicted to function as part of a backflow protection mechanism. Together these features are predicted to enable H^+^ pumps in vascular plants to create large electrochemical gradients that can be modulated in response to diverse physiological cues. The complete inventory of P-type ATPases in the major branches of Viridiplantae is an important starting point for elucidating the evolution in plants of these important pumps.

## Introduction

P-type ATPases are primary transporters energized by hydrolysis of ATP with a wide range of specificities for small cations and apparently also phospholipids (Møller et al., 1996; Palmgren and Harper, 1999). P-type ATPases are characterized by forming a phosphorylated intermediate (hence the name P-type), by being inhibited by vanadate, and by having a number of sequence motifs in common (Serrano, 1989; Axelsen and Palmgren, 1998). Plant P-type ATPases are characterized structurally by having a single catalytic subunit, 8–12 transmembrane segments, N and C termini exposed to the cytoplasm, and a large central cytoplasmic domain including the phosphorylation and ATP binding sites.

P-type ATPases constitute a large and indispensable family in most organisms. The P-type ATPase family can be divided into five major evolutionarily related subfamilies, P1–P5, which group in a phylogenetic tree according to the ions they transport (Axelsen and Palmgren, 1998). The P-type ATPases are involved in a wide range of fundamental cellular processes such as the efflux or organismal redistribution of micronutrients (P1B Zn^2+^- and Cu^2+^-ATPases), cellular signaling and Ca^2+^ compartmentalization (P2A and P2B Ca^2+^-ATPases), energizing the electrochemical gradient used as the driving force for the secondary transporters (P3A H^+^-ATPases in plants and fungi and P2C Na^+^/K^+^-ATPases in animals), and being involved in membrane vesicle budding (P4 ATPases). The function of P5 ATPases is not known but they have been implicated in vesicle budding from the endoplasmic reticulum (Poulsen et al., 2008a).

The bioenergetic systems of flowering plants and animals use different coupling ions (Skulachev, 1988; Rodríguez-Navarro, 2000). In animals, the potential energy that can be harvested to drive, e.g., nutrient transport across the plasma membrane derives from a Na^+^ gradient across this membrane. A Na^+^ pump in the plasma membrane is a very efficient system for extrusion of toxic Na^+^ (Gonzalez, 2011; Whittamore, 2012). A plasma membrane Na^+^ pump in the plasma membrane is also important for the extrusion of toxic Na^+^. Fish and invertebrates living in the salty oceans are dependent on such a pump for survival. For animals living in marine environments a Na^+^/K^+^ pumps (P2C ATPases) remain the sole system for formation of electrochemical ion gradients gradients in the plasma membrane (Morth et al., 2011).

In contrast, in plant and fungal cells, it is an electrochemical gradient of H^+^ that energizes the plasma membrane (Morth et al., 2011). In cells of flowering plants, the plasma membrane completely lacks Na^+^ pumps and depends entirely on plasma membrane H^+^ pumps for establishing a steep electrochemical ion gradient across the plasma membrane. In those flowering plants in which salt tolerance mechanisms have been investigated, strategies other than primary active extrusion of Na^+^ (i.e., efflux through a Na^+^ pump) appear to have evolved (Tester and Davenport, 2003; Flowers and Colmer, 2008). Unfortunately, most flowering plants remain very sensitive to saline environments and salinization of soils due to extensive irrigation is becoming an increasing problem for productivity of agriculture, especially in arid regions of the world (Tester and Davenport, 2003; Yamaguchi and Blumwald, 2005).

The apparent absence of Na^+^ pumps in flowering plants raises several questions. In ancestral plants containing both Na^+^ and H^+^ pumps, did both pumps help energize the plasma membrane, and did those membranes function with co-transport systems that could utilize both Na^+^ and H^+^ gradients? During the evolution of flowering plants, was there a physiological reason why plasma membranes might not function well with both Na^+^ and H^+^ pumps? Did the flowering plant lineage evolve from an ancestor living exclusively in a fresh water environment, or were Na^+^ pumps gradually lost at later points in evolution? Is it possible that Na^+^ pumps are still present in some flowering plants that have not yet been studied?

With the completion of genome sequences from all major branches of Viridiplantae, it is now possible to study the evolution of primary transport capabilities throughout the green plant lineage and to compare these transporters with those of protists, animals, and fungi. Our analysis of five selected plant genomes suggest that Na^+^ pumps coexisted with primitive plasma membrane H^+^ pumps in both early aquatic and terrestrial plants. However, at some point in the evolution of vascular plants, Na^+^ pumps appear to have been lost. The evolution of multicellular land plants also correlates with an expansion in number and potential regulatory features for H^+^ pumps, Ca^2+^ pumps, heavy metal pumps, and lipid flippases. In contrast, the P5 subfamily did not show an equivalent change, with only one or two isoforms present in all five plant lineages. This study provides an evolutionary framework for considering how P-type ATPases contribute to the biology of all Viridiplantae, from single celled algae to multicellular flowering plants.

## Materials and Methods

The P-type ATPase sequences were identified by searching for sequences in UniProtKB (The UniProt Consortium, 2012) and in Phytozome^1^ from the relevant genomes that matched the PFAM profile PF00122 and in UniProtKB also the PROSITE pattern PS00154 (DKTG[T,S][L,I,V,M][T,I]; Axelsen and Palmgren, 1998). The PROSITE pattern is unique for P-type ATPases while the PFAM profile is more inclusive and also matches sequences that cannot function as active P-type ATPases as they lack the phosphorylated Asp residue that is present in the PROSITE pattern. Since the sequences in Phytozome do not include information about matches to PROSITE patterns, only PFAM was used to select sequences from this resource. The identified sequences were aligned and duplicates were removed. All genomes except *Arabidopsis* are still draft versions in the databases, so often the predicted P-type ATPases do not represent complete proteins. Furthermore, the versions identified in UniProtKB and in Phytozome were not 100% identical. When possible, the UniProtKB sequence was chosen.

The resulting dataset of 150 sequences were aligned using Muscle (Edgar, 2004). The resulting alignment of the 150 full-length sequences was used to construct a phylogenetic tree using the Neighbor Joining method as implemented in QuickTree (Howe et al., 2002). The standard parameters of Muscle and QuickTree were used. For visualization of the constructed tree we used Dendroscope (Huson et al., 2007). A phylogenetic tree for each subgroup (P1–P5) of the 150 sequences was constructed and visualized similarly. For each subgroup, the corresponding set of sequences was selected from the dataset of 150 sequences. The selected sequences and a few additional outlier sequences were aligned using Muscle and a phylogenetic tree was constructed using QuickTree. The trees for the individual subfamilies are all rooted with the sequence of the *E. coli* P1A ATPase KdpB (P03960) as outgroup. The 150 sequences as well as sequences for the outliers employed are available as Fasta files in Supplementary Information.

## Results

**Figure 1:**
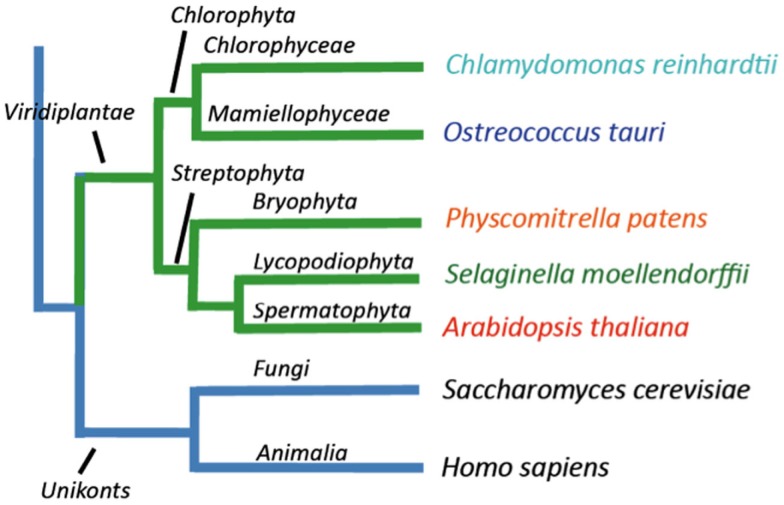
**Overview of the evolutionary relationship between the green plant genomes analyzed in this study**.

In this work we identified genes encoding P-type ATPases in the sequenced genomes from five representatives of the green plant lineage (Viridiplantae), which previously diverged from opisthokonts (animals, fungi, and Choanozoa; Yoon et al., 2004; Figure 1). The Chlorophytae (green algae, including *Chlamydomonas* and *Ostreococcus*) diverged from the Streptophytae (land plants and their close relatives; Figure 1) over a billion years ago. In this work, the genomes of two green algae were analyzed, namely those of *Ostreococcus tauri* (Derelle et al., 2006) and *Chlamydomonas reinhardtii* (Merchant et al., 2007), and three land plants, the moss *Physcomitrella patens* (Rensing et al., 2008), the primitive vascular plant *Selaginella moellendorffii*^2^ and the flowering plant *Arabidopsis thaliana* (The Arabidopsis Genome Initiative, 2000). The latter has previously been investigated for its content of P-type ATPases (Axelsen and Palmgren, 2001).

*Ostreococcus tauri* is an extremely small (0.8 μm wide) unicellular green alga, which belongs to the Prasinophyceae, one of the most ancient groups within the lineage of green plants (Courties et al., 1998). This organism is a naked, non-flagellated cell possessing a single mitochondrion and a single chloroplast, and a common member of global oceanic picoplankton populations. *C. reinhardtii* is a much bigger (∼10 μm), likewise unicellular green alga. It lives in terrestrial soils and has multiple mitochondria, two anterior flagella for motility and mating, and a chloroplast (Rochaix, 1995). *P. patens* is a moss, a representative for primitive land plants without a vascular system. *S. moellendorffii* is a member of an ancient vascular plant lineage that first appears in the fossil record about 400 million years ago (Banks, 2009). *A. thaliana* was the first angiosperm to have its genome sequenced, and is a model plant for understanding the molecular biology of flowering plants.

**Figure 2:**
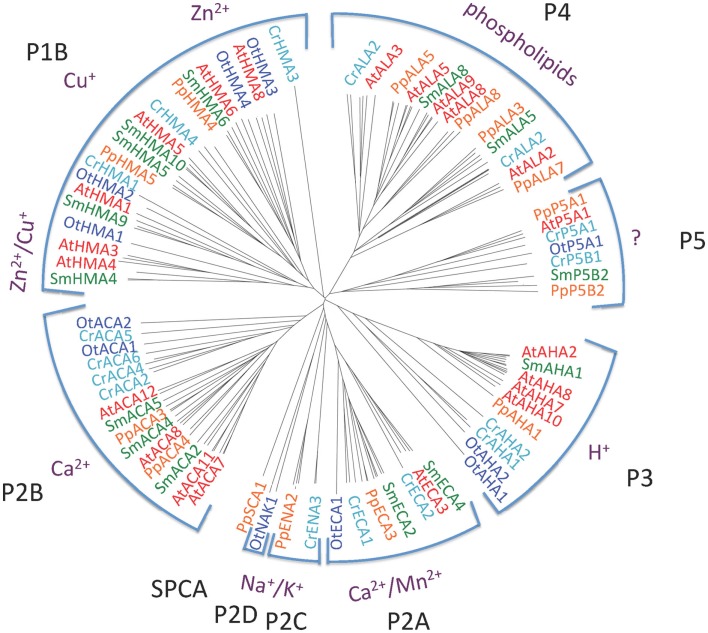
**Phylogenetic tree of P-type ATPases analyzed in this study**. Not all branches are labeled. Accession numbers for sequences are given in Table 2.

Figure 2 shows the phylogenetic relationship of all the 150 sequences listed in Table 2, which were evaluated in this study. They are distributed in all five major families of P-type ATPases with some noteworthy comparisons discussed below.

### P1B ATPases: Heavy metal pumps

Heavy metal pumps (P1B ATPases) are found in all life forms including bacteria. In eukaryotes, these pumps are typically encoded by multigene families (Axelsen and Palmgren, 1998). The model dicotyledonous plant *A. thaliana* contains eight P1B ATPases, which can be divided into three groups according to conserved sequence motifs and their putative substrate specificity (Axelsen and Palmgren, 2001; Williams and Mills, 2005; Argüello et al., 2007). AtHMA5 to 8 belong to group P1B-1 and are predicted to transport Cu/Ag, while AtHMA2 to 4 belong to group P1B-2 predicted to transport Zn/Cd, and AtHMA1 belongs to group P1B-4 with a predicted broad substrate specificity (Zn/Cu/Co/Cd/Pb/Ca).

**Figure 3:**
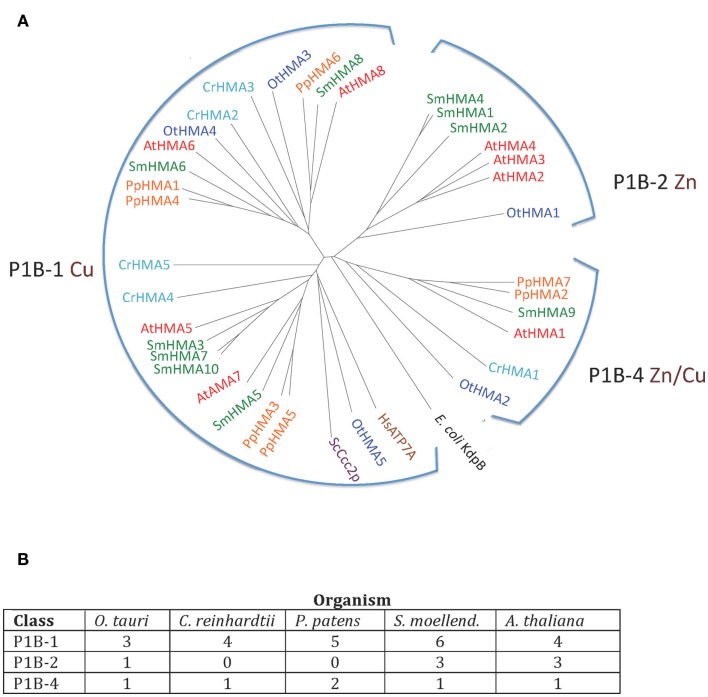
**(A)** Phylogenetic tree of P1B ATPases (heavy metal pumps) from Viridiplantae. Accession numbers for sequences are given in Table 2. For comparison, the following outliers were included: a bacterial P1A ATPase *E. coli* KdpB (P03960), and HsATP7A (Q04656), and ScCcc2p (P38995), Cu^+^ pumps from *H. sapiens* and *S. cerevisiae*, respectively. **(B)** Numbers of P1B ATPases by subgroups (Argüello et al., 2007) in the five plant genomes analyzed.

P1B ATPases were found in all organisms investigated in this study (Figure 3). The genome of *C. reinhardtii* encodes five P1B ATPases, four of which (CrHMA2-5) belong to the P1B-1 cluster. A representative protein in *A. thaliana* from this cluster is AtHMA8/PAA2. This protein localizes to the thylakoid membrane of chloroplasts and is required for Cu delivery during the biogenesis of plastocyanin (Abdel-Ghany et al., 2005).

The only heavy metal ATPase in *C. reinhardtii* that is not a P1B-1 pump, is CrHMA1, which belongs to the P1B-4 cluster in which AtHMA1 is also found. AtHMA1 is localized to the chloroplast inner envelope membrane (Seigneurin-Berny et al., 2006; Kim et al., 2009). This ATPase has been reported to function as a transporter for Cu and Ca in addition to Zn/Cd/Co (Seigneurin-Berny et al., 2006; Moreno et al., 2008; Kim et al., 2009). All five organisms investigated in this study had one or two representatives of P1B-4 ATPases.

**Figure 4:**
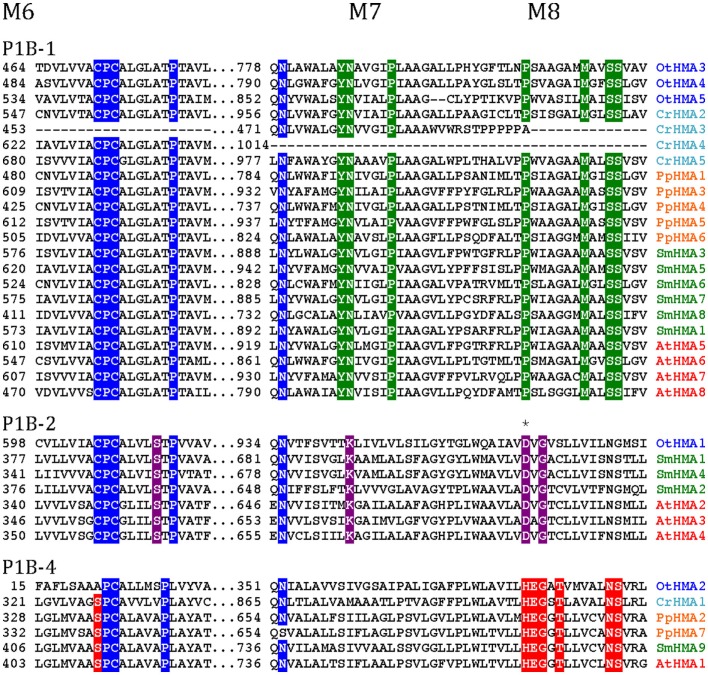
**Alignment of three transmembrane segments from Viridiplantae P1B ATPases analyzed in this study**. Only the predicted transmembrane segments M6, M7, and M8 are shown. P1B-1: Cu^+^ transporting ATPases; P1B-2: Zn^2+^ transporting ATPase: P1B-4: Mixed specificity heavy metal pumps. The *asterisk* indicates an Asp residue (D) conserved in P1B-2 ATPases, which could be important for coordination of Zn^2+^ (Dutta et al., 2006).

Zn transporting P1B ATPases are common in prokaryotes but have not been identified in fungi and animals. In *O. tauri*, which has one of the smallest known eukaryotic genomes, there are five P1B ATPases (∼30% of its P-type ATPase genes), which distribute among all three clusters of P1B ATPases, including a single P1B-2 pump. In our analysis, P1B-2 ATPases were found in *O. tauri* (OtHMA1), *S. moellendorffii* (SmHMA1, SmHMA2, and SmHMA4), and *A. thaliana* (AtHMA2, AtHMA3, and AtHMA4; Figures 3 and 4). P1B-2 ATPases could not be identified in *C. reinhardtii* and *P. patens*. Among the *A. thaliana* P1B-2 ATPases, AtHMA2, and AtHMA4 are localized to the plasma membrane. They show redundant function in cellular export of Zn and Cd into plant vascular tissues where they facilitate xylem loading and transport to the shoot (Hussain et al., 2004; Verret et al., 2004; Mills et al., 2003; Wong and Cobbett, 2009).

As P1B-2 Zn ATPases are absent from fungi and animals, but common in bacteria, it seems likely that in plants these pumps have evolved from chloroplastic pumps. With the advent of vascular plants, it is likely that a subset of P1B-2 ATPases was targeted to the plasma membrane and acquired a new role in redistribution of Zn within the plant body.

### P2A ATPases: ER-type Ca^2+^-ATPases

**Figure 5:**
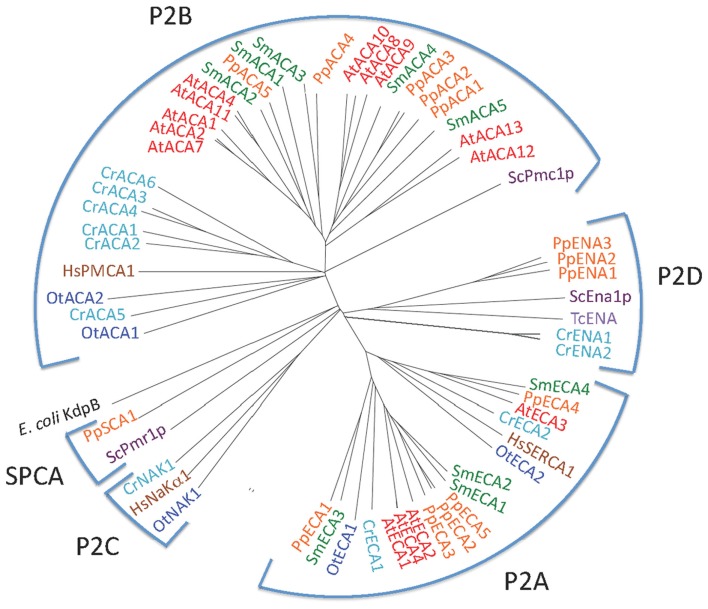
**Phylogenetic tree of P2 ATPases (Ca^2+^ and Na^+^ pumps)**. Accession numbers for sequences are given in Table 2. For comparison, the following outliers were included: a bacterial P1A ATPase *E. coli* KdpB (P03960), the *S. cerevisiae* pumps ScPmr1p (P13586), ScPmc1p (P38929), and ScEna1p (P13857), the *H. sapiens* pumps HsPMCA1 (P20200), HsSERCA1 (O14983), and HsNaKα1 (P98194), and the *Trypanosoma cruzi* pump TcENA (Q76DT8).

P2 ATPases form a large subfamily further divided into at least four clusters of pumps, two (P2A and P2B) having specificity for Ca^2+^ and two (P2C and P2D) for Na^+^ as the transported ligand (Figure 5).

P2A ATPases were identified in all plants studied here (Figure 5). A single pump from each organism (OtECA2, CrECA4, PpECA4, SmECA4, and AtECA3) form a distinct subset of ER-type Ca^2+^-ATPases (ECAs) closely related to the animal sarco-endoplasmic reticulum Ca^2+^ pump SERCA1 (Figure 5). In the flowering plant *A. thaliana*, AtECA3 is a Golgi-localized pump that can transport Ca^2+^ and Mn^2+^ (Li et al., 2008; Mills et al., 2008).

**Figure 6:**
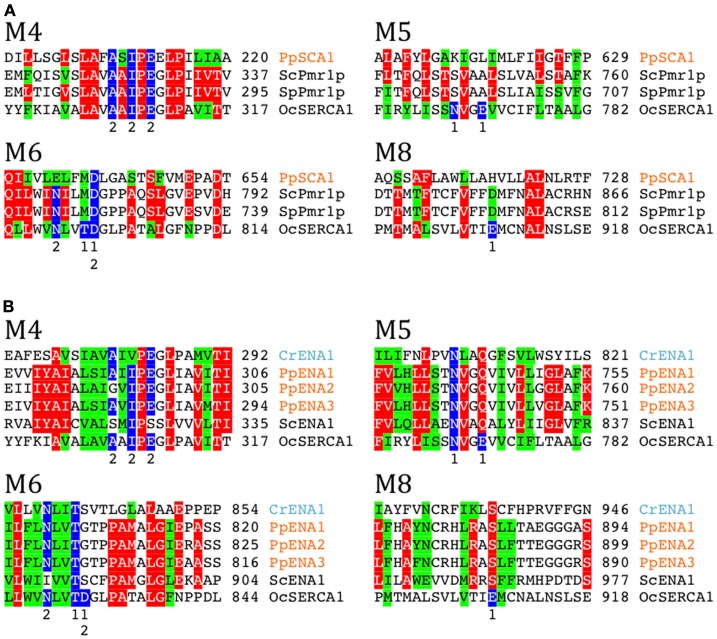
**Alignment of transmembrane segments showing differences in a potential cation binding site for selected Ca^2+^ and Na^+^ pumps**. Secretory pathway Ca^2+^ATPases (SPCA pumps) and ENA Na^+^ pumps (P2D ATPases) are missing Ca^2+^ binding site 1 present in P2A pumps typified by rabbit OcSERCA1 (P04191). In P2A pumps a conserved Asp in M6 contributes to coordination of both Ca^2+^ ions. **(A)** Secretory pathway pumps (SPCAs) have a conserved Asp in M6. Fungal examples shown are *S. cerevisiae* Pmr1p (P13586) and *S. pombe* Pmr1p (O59868). **(B)** P2D ATPases (ENA pumps) do not have a conserved Asp in M6 like all other P2 ATPases. Residues contributing with Ca^2+^ coordinating oxygen molecules in rabbit SERCA1 (Toyoshima et al., 2000) are marked in blue. Only sequences including the predicted transmembrane segments M4, M5, M6, and M8 are shown.

In a comparison among many eukaryotes, a subset of P2A Ca^2+^-ATPases form a distinct cluster and have been named secretory pathway Ca^2+^-ATPases (SPCAs; Wuytack et al., 2003). These pumps are identified in fungal and animal cells and are localized to the Golgi apparatus or other membranes of the secretory pathway. As evident from Figure 6, representatives of these pumps from fungi (*S. cerevisiae* and *S. pombe*) are characterized by having lost Ca^2+^ binding site 1.

Only a single likely SPCA protein was found in our analysis, namely PpSCA1. This protein clusters with other SPCAs and does not contain conserved residues expected for Ca^2+^ binding site 1 (Figure 6A). Among reference plant and animal genomes surveyed, PpSCA1 showed the greatest identity (30%) to the secretory pathway Ca^2+^-ATPase Pmr1p from *S. cerevisiae* (Rudolph et al., 1989; Antebi and Fink, 1992).

### P2B ATPases: Autoinhibited Ca^2+^-ATPases

P2B ATPases are Ca^2+^ pumps that are activated by binding of calmodulin to autoinhibitory terminal domains. A marked difference between higher plant and animal P2B ATPases is that their calmodulin-binding domains (CMBDs) are situated in the N- and C-terminal domains, respectively (Sze et al., 2000). Autoinhibited Ca^2+^-ATPases (ACAs) from flowering plants have been shown to be activated by Ca^2+^ in the presence of calmodulin and are characterized by an N-terminally situated CMBD (Malmström et al., 1997; Harper et al., 1998; Curran et al., 2000; Hwang et al., 2000). The CMBD overlaps partially with an autoinhibitory pump sequence (Bækgaard et al., 2006) and it has been proposed that calmodulin, by binding to the CMBD, neutralizes the constraint set by the autoinhibitory sequence on the pump molecule (Bækgaard et al., 2006). Animal P2B ATPases are likewise activated by calmodulin, but in these the CMBD is situated in an extended C-terminal domain (James et al., 1988).

**Figure 7:**
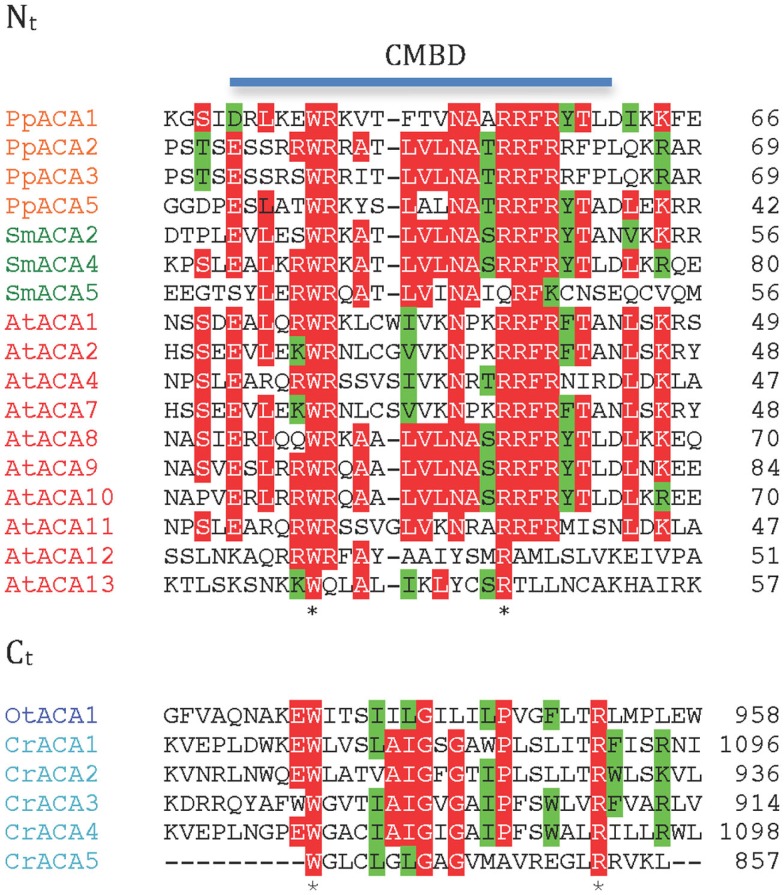
**Alignment of putative calmodulin-binding sites in the N- and C-terminal regions of ACA pumps**. Nt, N-terminal domain; Ct, C-terminal domain.

Putative CMBDs were identified in the N-terminal domains of most P2B ATPases of *P. patens*, *S. moellendorffii*, and *A. thaliana* (Figure 7). The proposed CMBDs in AtACA12 and AtACA13 are weakly defined. Importantly, an N-terminal CMBD could not be identified in P2B ATPases from the chlorophytes *O. tauri* and *C. reinhardtii*.

As animal P2B ATPases are equipped with a C-terminally located CMBD, we analyzed all of the plant P2B ATPases for a putative regulatory domain in this location. CMBDs have very little similarity between calmodulin-binding proteins and can be difficult or impossible to predict with certainty although as a rule there is alternation between bulky aromatic and positively charged residues. No C-terminal extensions could be identified in Streptophyte P2B ATPases. In contrast, a conserved sequence with weak resemblance of a CMBD was identified in the C-terminal domain of P2B ATPases from the chlorophytes *O. tauri* and *C. reinhardtii* (Figure 7), which are missing a similar sequence from their N-terminal domain. The calmodulin-binding capacity of these putative CMBDs remains to be tested, but it is an attractive working hypothesis that swapping of the CMBD from the C-terminal to the N-terminal domain occurred at the split between Chlorophytae and Streptophytae.

### P2C ATPases: Na^+^/K^+^-ATPases

The Na^+^/K^+^-ATPase of animal cells was the first P-type ATPase to be discovered (Skou, 1957), but all subsequent attempts to identify a Na^+^ pump in vascular plants failed. Vascular plants tend to be very sensitive to elevated Na^+^ in the soil, which is probably due to the lack of an effective Na^+^ extrusion system such as a Na^+^/K^+^-ATPase. In animal cells, the plasma membrane is energized by the Na^+^/K^+^-ATPase whereas in a typical plant, the plasma membrane H^+^-ATPases (P3A ATPases) carry out this function. It has therefore been hypothesized that P2C Na^+^/K^+^-ATPases were lost at a branch point in the Streptophyta plant lineage, presumably in an organism that evolved in a fresh water environment and utilized a plasma membrane H^+^-ATPase to energize its plasma membrane (Palmgren, 2001).

**Figure 8:**
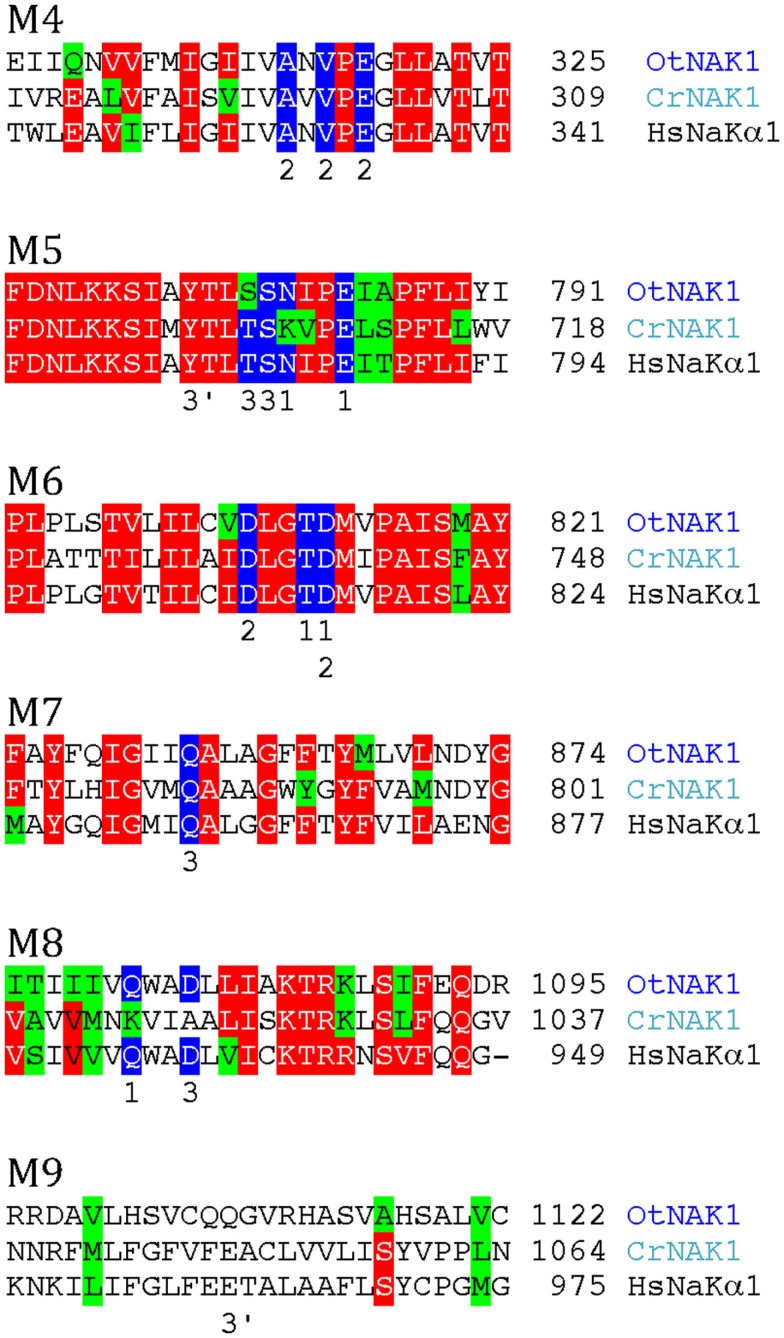
**Alignment of predicted transmembrane segments of putative chlorophyte Na^+^/K^+^-ATPases (OtNAK1 and CrNAK1) with similar regions in the human Na^+^/K^+^-ATPase α1 subunit (ATP1A1; P05023)**. Residues that in Na^+^/K^+^-ATPase are likely to contribute with oxygen atoms for coordination of Na^+^ (Morth et al., 2007) are marked by blue. Numbers above these residues refer to which of the three Na^+^ sites (1–3) they contribute to. Residues contributing to an alternative Na^+^ site (site 3b) are marked 3′.

Biochemical evidence has pointed to the presence an electrogenic, vanadate-sensitive, ouabain-resistant Na^+^-ATPase in the plasma membrane of marine chlorophytes (Popova et al., 1999, 2005; Gimmler, 2000) and chlorophyte expressed sequence tags with similarity to Na^+^/K^+^-ATPase have been identified (Barrero-Gil et al., 2005). Both chlorophytes analyzed in this work contain sequences with strong similarity to an animal Na^+^/K^+^-ATPase (Figure 8). No Na^+^/K^+^-ATPase has been crystallized in a form with bound Na^+^, but a homology model has been built (Morth et al., 2011) based on the structure of a Na^+^/K^+^-ATPase with bound Rb^+^ (as a substitute for K^+^) and the structure of SERCA1 with bound Ca^2+^. According to this model, residues in several transmembrane segments of Na^+^/K^+^-ATPase contribute with binding ligands to Na^+^ (Figure 8). All these residues are conserved in OtNAK1 and CrNAK1, strongly suggesting that these ATPases operate as Na^+^ pumps. *O. tauri* is a chlorophyte that lives in oceans, where a Na^+^/K^+^-ATPase is of obvious benefit for extrusion of Na^+^ leaking in from sea water. However, it is peculiar that *C. reinhardtii*, a green alga of terrestrial soils, is also equipped with such a pump. This suggests that the presence of Na^+^/K^+^ pumps in green algae is a primitive character that was lost with the emergence of Streptophytae. This hypothesis is supported by the widespread presence of P2C pumps in other eukaryotes and archaea (Sáez et al., 2009).

As mentioned above, the substrate specificity of PpSCA1 is uncertain, with features that are both consistent and contrary to speculations on the transport of either Na^+^ or Ca^2+^. This pump does have some similarity with chlorophyte Na^+^/K^+^-ATPases, but ligands contributing to Na^+^ Site 3 are not present and similarity to sites contributing to sites 1 and 2 are not absolute (Figure 6A). Biochemical and genetic experiments are needed to address the question of substrate specificity for PpSCA1.

### P2D ATPases: Na^+^ or K^+^ pumps of mosses and fungi

P2D ATPases form a unique group of pumps so far only found in mosses, fungi, and protozoa and confer Na^+^ tolerance to organisms in which they are expressed (Rodríguez-Navarro and Benito, 2010). The moss *P. patens* encodes three P2D ATPases: PpENA1, PpENA2, and PpENA3. Related pumps are present in liverworts (Marchantiophyta), such as *Marchantia polymorpha* and *Riccia fluitans*, which are primitive non-vascular land plants related to mosses. A close fungal homolog to PpENA1 is from *Neurospora crassa* (Q9UUX8; 43% identity). When protein databases were searched for similar proteins outside plants and fungi, hits were only found in protist sequences.

In P2A ATPases, two Ca^2+^ sites are present: Site 1 and Site 2 (Toyoshima et al., 2000). In SPCAs (Figure 6A) and P2D ATPases (Figure 6B), Site 1 is missing. Thus, two negatively charged Glu residues in M6 and M8, which in SERCA1 contribute to Ca^2+^ coordination in Site 1, are absent in both secretory pathway pumps and P2D pumps. Notably, a negatively charged Asp in M6, which is conserved in all other P2-type ATPases, is replaced by a neutral residue in P2D pumps (Rodríguez-Navarro and Benito, 2010; Figure 6B). In the available structures of P2A, P2C, and P3A ATPases (Morth et al., 2007; Olesen et al., 2007; Pedersen et al., 2007), this Asp contributes to coordination of all transported cations including Ca^2+^, Na^+^, K^+^, and H^+^. The absence of the Asp in M6, in addition to other negatively charged amino acid residues in the membrane domain, therefore appears to be a hallmark of P2D ATPases. When the sequences of P-type ATPases retrieved in this study were analyzed in detail it appeared that two pumps from *C. reinhardtii*, here named CrENA1 and CrENA2, are likely to represent chlorophyte P2D ATPases as they lack a negatively charged Asp in M6 (Figure 6B). Although these branch out close to P2D pumps (Figure 5) they have less than 33% identity to these or any other plant pumps.

### P3A ATPases: Autoinhibited H^+^-ATPases

**Figure 9:**
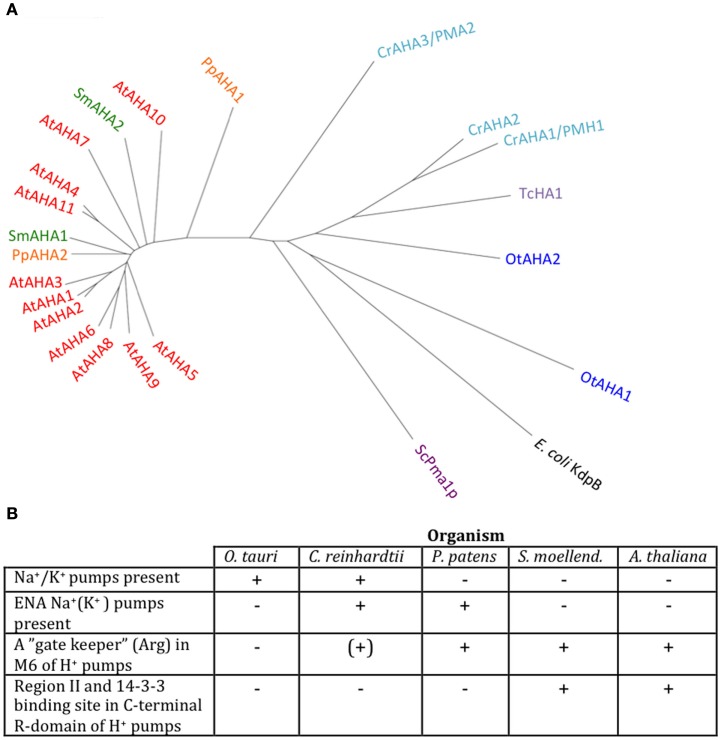
**Phylogenetic tree of P3 ATPases (H^+^-pumps) (A) and table summarizing conserved features (B)**. Accession numbers for sequences are given in Table 2. For comparison, the following outliers were included: a bacterial P1A ATPase *E. coli* KdpB (P03960), the *S. cerevisiae* pump ScPma1p (P05030), and the *Trypanosoma cruzi* pump TcHA1 (Q8T7V7).

We identified P3A ATPases in all genomes of Viridiplantae analyzed in this work (Figure 9). P3A ATPases energize the plasma membrane of plants and fungi by establishing a large proton gradient and membrane potential (negative on the inside) across the plasma membrane (Palmgren, 2001). The potential energy stored in this gradient serves as a proton motive force that drives a large number of transport processes carried out by secondary active transporters and channel proteins. The plasma membrane proton pump isoform 2 from *A. thaliana* (autoinhibited H^+^-ATPase 2, AHA2) is expressed throughout the plant and, together with the closely related isoform AHA1, is essential for plant growth (Palmgren, 2001; Haruta et al., 2010). In this respect, they serve as functional analogs to the Na^+^/K^+^-ATPases of animal cells (Morth et al., 2011).

In *Chlamydomonas*, two plasma membrane H^+^-ATPases have been described in the literature (Campbell et al., 2001). Closely related homologs to OtAHA2 are found in other green algae but not in streptophytes. Outside this group, OtAHA2 has highest similarity (42–44% identity) to plasma membrane H^+^-ATPases of protists that have been characterized biochemically as P-type H^+^-ATPases (Luo et al., 2002, 2006; Figure 9). OtAHA2 has lesser but marked similarity to AtAHA2 (38% identity). The most divergent P3A ATPase analyzed in this study is OtAHA1.

**Figure 10:**
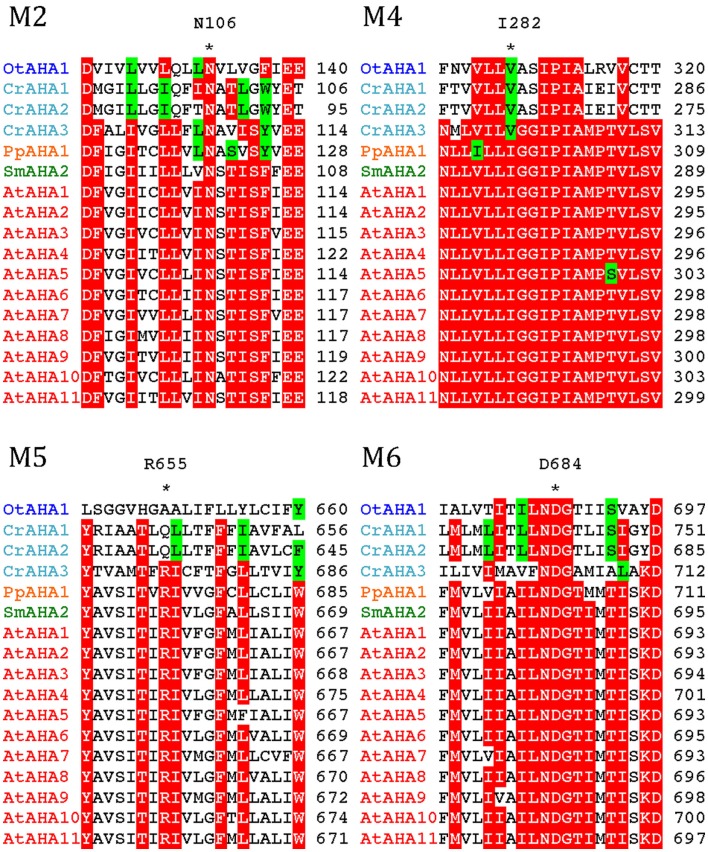
**Alignment of predicted transmembrane segments in P3A H^+^-ATPases**. Asterisks mark residues of potential importance for proton coordination and pumping based on evidence from mutagenesis and analysis of a crystal structure for AtAHA2 (Pedersen et al., 2007). The number of the amino acid residue in AtAHA2 is indicated above each asterisk. R655 in M5, which seems important for controlling backflow of H^+^ at high electrochemical gradients (Pedersen et al., 2007), is lacking in some chlorophyte H^+^-ATPases.

All P3A plasma membrane H^+^-ATPases have conserved residues that have been implicated as being important for H^+^ transport (Pedersen et al., 2007; Figure 10). These include the H^+^ acceptor/donor Asp684 (AtAHA2 numbering) in M6 and the proposed gate-keeper residue Asn106 in M2 (Pedersen et al., 2007; Buch-Pedersen et al., 2009). Arg655, which in AtAHA2 has been proposed to prevent backflow of H^+^ through the pump, a feature likely to be essential when electrochemical gradients get steep, is strictly conserved in all streptophyte pumps. Interestingly, this residue is absent in chlorophyte P3A ATPases except for CrAHA3 (Figure 10). CrAHA3 is the chlorophyte pump that shows the highest similarity to a streptophyte H^+^ pump (Figure 9).

Notably, typical protists and chlorophytes are characterized by having both Na^+^ and H^+^ pumps. Protists are equipped with P2D Na^+^ pumps and chlorophytes with P2C Na^+^/K^+^ pumps (Table 1; Figures 8 and 9). As plasma membrane H^+^-ATPases in these organisms lack the residue corresponding to Arg655 (to block H^+^ backflow), this would suggest that in organisms with co-expression of electrogenic H^+^ and Na^+^ pumps, it is the role of Na^+^ ATPases to generate a plasma membrane electrochemical gradient, which in turn can be used as an energy source to drive a variety of cellular processes, such as secondary active transport. P3A ATPases in protists and chlorophytes might therefore have other roles than establishing electrochemical gradients, e.g., controlling intracellular pH.

**Table 1:** **Overview of number of pumps in different P-type ATPase subfamilies in Viridiplantae**.

Class	Organism					Total
	*O. tauri*	*C. reinhardtii*	*P. patens*	*S. moellendorffii*	*A. thaliana*	
P1B	5	5	7	10	8	35
P2A	2	2	5	4	4	17
P2B	2	6	6	5	10	29
P2C	1	1	0	0	0	2
P2D	0	2	3	0	0	5
P3A	2	3	2	2	11	20
P4	1	3	8	8	12	32
P5A	1	1	1	1	1	5
P5B	0	1	2	2	0	5
Total	14	24	34	32	46	150

**Table 2:** **List of P-type ATPases analyzed in this study**.

Class	Gene	aa	Accession #	Locus/ORF
***OSTREOCOCCUS TAURI***				
P1B	OtHMA1	1052	Q01EZ3	Ot02g01930
P1B	OtHMA2	681	Q00V55^1^	Ot15g01530
P1B	OtHMA3	1359	Q014R9	Ot07g03370
P1B	OtHMA4	861	Q00YQ6	Ot11g02480
P1B	OtHMA5	925	Q018N8	Ot05g03820
P2A	OtECA1	561	Q01FX0^1^	Ot01g04600
P2A	OtECA2	1013	Q01C29	Ot03g04740
P2B	OtACA1	1062	Q011R1	Ot09g02100
P2B	OtACA2	780	Q00U35^1^	Ot16g02150
P2C	OtNAK1	1172	Q01GZ3	Ot01g00900
P3A	OtAHA1	1043	Q00RY3^2^	Ot20g00330
P3A	OtAHA2	879	Q017J6^3^	Ot06g01560
P4	OtALA1	1258	Q016N2	Ot06g03680
P5A	OtP5A1	1398	Q00 × 11	Ot13g01720
***CHLAMYDOMONAS REINHARDTII***				
P1B	CrHMA1	1189	A8J6D5	CHLREDRAFT_195998
P1B	CrHMA2/CTP2	1086	A8IC93	CHLREDRAFT_205938
P1B	CrHMA3/CTP3	504	A8ICL1^4^	CHLREDRAFT_165985
P1B	CrHMA4/CTP1	1097	A8JBB5	CHLREDRAFT_206047
P1B	CrHMA5	1041	A8J829	CHLREDRAFT_195962
P2A	CrECA1	1069	A8I542	CHLREDRAFT_77047
P2A	CrECA2	845	g762350^5^,^6^	Cre22.g762350
P2B	CrACA1	1179	A8IZL7	CHLREDRAFT_196702
P2B	CrACA2	1009	A8IJV9	CHLREDRAFT_128099
P2B	CrACA3/FAB39	930	A8J0V2^1^	CHLREDRAFT_118223
P2B	CrACA4	1191	A8IS11	CHLREDRAFT_189266
P2B	CrACA5	873	A8JEM4^7^	CHLREDRAFT_179136
P2B	CrACA6	1434	A8HM60/g036350^8^	CHLREDRAFT_116583/Cre01.g036350
P2C	CrNAK1	1112	A8HX15	CHLREDRAFT_187139
P2D	CrENA1	1004	A8J4M6	CHLREDRAFT_104709
P2D	CrENA2	1001	A8J4M4	CHLREDRAFT_130931
P3A	CrAHA1/CrPMH1	1081	A8IFK0/Q9FNS3	CHLREDRAFT_54949
P3A	CrAHA2	802	A8IFH0^1^	CHLREDRAFT_38137
P3A	CrAHA3/CrPMA2	1081	Q93Z22	CHLREDRAFT_182602
P3A	–	1628	g316950^9^	Cre07.g316950
P4	CrALA1	1183	A8IVJ3	CHLREDRAFT_172401
P4	CrALA2	1300	A8IVJ6	CHLREDRAFT_190292
P4	CrALA3	1243	A8J8G9^6^	CHLREDRAFT_193025
P5A	CrP5A1	1168	A8JI26^1^	CHLREDRAFT_60708
P5B	CrP5B1	1308	A8I9J2^1^	CHLREDRAFT_186680
***PHYSCOMITRELLA PATENS***				
P1B	PpHMA1	902	A9RNK6	PHYPADRAFT_117222
P1B	PpHMA2	743	A9S8B9^1^	PHYPADRAFT_125638
P1B	PpHMA3	1004	A9T8Q3	PHYPADRAFT_192723
P1B	PpHMA4	841	A9SUQ2	PHYPADRAFT_215914
P1B	PpHMA5	1009	A9SME3	PHYPADRAFT_81365
P1B	PpHMA6	893	A9TB46	PHYPADRAFT_142921
P1B	PpHMA7	743	A9TQB5	PHYPADRAFT_148958
P2A	PpECA1	1060	A9RZK8	PHYPADRAFT_179791
P2A	PpECA2	1039	A9SH40	PHYPADRAFT_184915
P2A	PpECA3	1055	A9SHQ4	PHYPADRAFT_212461
P2A	PpECA4	1000	A9TIL4	PHYPADRAFT_222630
P2A	PpECA5	1037	A9TK26	PHYPADRAFT_223041
P2A	PpSCA1	822	A9T9F0	PHYPADRAFT_220109
P2B	PpACA1/PCA1	1098	Q70TF0	PHYPADRAFT_202276
P2B	PpACA2/PCA2	1105	Q70TF1	PHYPADRAFT_224496
P2B	PpACA3	1074	A9SLT6	PHYPADRAFT_230135
P2B	PpACA4	948	A9RXA7^10^	PHYPADRAFT_121055
P2B	PpACA5	1035	A9RZJ8	PHYPADRAFT_121834
P2D	PpENA1	967	Q7XB51	
P2D	PpENA2	1058	Q7XB50	
P2D	PpENA3	963	C1L359	PHYPADRAFT_112089
P3A	PpAHA1	936	A9U0N9	PHYPADRAFT_153928
P3A	PpAHA2	465	A9TU44^1^	PHYPADRAFT_198308
P4	PpALA1	1219	A9RVW0	PHYPADRAFT_205967
P4	PpALA2	1062	A9S030	PHYPADRAFT_121975
P4	PpALA3	1229	A9S076	PHYPADRAFT_122321
P4	PpALA4	1194	A9SKC3	PHYPADRAFT_165384
P4	PpALA5	1251	A9SY94	PHYPADRAFT_189702
P4	PpALA6	1262	A9T6U6	PHYPADRAFT_88857
P4	PpALA7	1104	A9T776	PHYPADRAFT_168461
P4	PpALA8	1151	A9TDQ8	PHYPADRAFT_221270
P5A	PpP5A1	1178	A9RG70	PHYPADRAFT_113743
P5B	PpP5B1	435	A9TN06^1^	PHYPADRAFT_147950
P5B	PpP5B2	1290	A9SGC2	PHYPADRAFT_229474
***SELAGINELLA MOELLENDORFFII^11^***				
P1B	SmHMA1	819	D8RBL1	SELMODRAFT_89397
P1B	SmHMA2	696	D8QRZ3^1^	SELMODRAFT_60690
P1B	SmHMA3	952	D8SD62	SELMODRAFT_114297
P1B	SmHMA4	831	D8SJM4	SELMODRAFT_118425
P1B	SmHMA5	1018	D8SPX5	SELMODRAFT_122320
P1B	SmHMA6	910	D8TEP8	SELMODRAFT_138129
P1B	SmHMA7	953	D8RFP0	SELMODRAFT_92276
P1B	SmHMA8	790	D8QYH6^1^	SELMODRAFT_60775
P1B	SmHMA9	817	D8R2L0	SELMODRAFT_167322
P1B	SmHMA10	960	D8R2W8	SELMODRAFT_84115
P2A	SmECA1	1047	D8RSK1	SELMODRAFT_267711
P2A	SmECA2	1042	D8RUH8	SELMODRAFT_102055
P2A	SmECA3	1045	D8SA88	SELMODRAFT_112465
P2A	SmECA4	1009	D8SUV3	SELMODRAFT_158488
P2B	SmACA1	1014	D8QTC2	SELMODRAFT_437746
P2B	SmACA2	1030	D8QTC3	SELMODRAFT_266601
P2B	SmACA3	1068	D8T1F8	SELMODRAFT_129812
P2B	SmACA4	1105	D8S012	SELMODRAFT_451372
P2B	SmACA5	1069	D8T4Q6	SELMODRAFT_451597
P3A	SmAHA1	875	D8T8I0	SELMODRAFT_430150
P3A	SmAHA2	952	D8QT85^1^	SELMODRAFT_76771
P4	SmALA1	1207	D8QMQ3	SELMODRAFT_164122
P4	SmALA2	1157	D8R8G6	SELMODRAFT_86830
P4	SmALA3	1109	D8RG22	SELMODRAFT_410847
P4	SmALA4	1009	D8RKR6	SELMODRAFT_95836
P4	SmALA5	1208	D8S239	SELMODRAFT_107016
P4	SmALA6	1221	D8SBS1	SELMODRAFT_113552
P4	SmALA7	1153	D8SGU6	SELMODRAFT_116847
P4	SmALA8	1184	D8TF22	SELMODRAFT_138337
P5A	SmP5A1	1109	D8TBK6	SELMODRAFT_187116
P5B	SmP5B1	1290	D8SFE9	SELMODRAFT_445079
P5B	SmP5B2	1246	D8R5P0	SELMODRAFT_439526
***ARABIDOPSIS THALIANA***				
P1B	AtHMA1	819	Q9M3H5	At4g37270
P1B	AtHMA2	951	Q9SZW4	At4g30110
P1B	AtHMA3	760	P0CW78	At4g30120
P1B	AtHMA4	1172	O64474	At2g19110
P1B	AtHMA5	995	Q9SH30	At1g63440
P1B	AtHMA6/PAA1	949	Q9SZC9	At4g33520
P1B	AtHMA7/RAN1	1001	Q9S7J8	At5g44790
P1B	AtHMA8/PAA2	856	Q9C594	At5g21930
P2A	AtECA1	1061	P92939	At1g07810
P2A	AtECA2	1054	O23087	At4g00900
P2A	AtECA3	998	Q9SY55	At1g10130
P2A	AtECA4	1061	Q9XES1	At1g07670
P2B	AtACA1	1020	Q37145	At1g27770
P2B	AtACA2	1014	O81108	At4g37640
P2B	AtACA4	1030	O22218	At2g41560
P2B	AtACA7	1015	O64806	At2g22950
P2B	AtACA8	1074	Q9LF79	At5g57110
P2B	AtACA9	1086	Q9LU41	At3g21180
P2B	AtACA10	1069	Q9SZR1	At4g29900
P2B	AtACA11	1025	Q9M2L4	At3g57330
P2B	AtACA12	1033	Q9LY77	At3g63380
P2B	AtACA13	1017	Q9LIK7	At3g22910
P2B	–	1049	F4KHQ2^12^	At5g53010
P3A	AtAHA1	949	P20649	At2g18960
P3A	AtAHA2	948	P19456	At4g30190
P3A	AtAHA3	949	P20431	At5g57350
P3A	AtAHA4	960	Q9SU58	At3g47950
P3A	AtAHA5	949	Q9SJB3	At2g24520
P3A	AtAHA6	949	Q9SH76	At2g07560
P3A	AtAHA7	961	Q9LY32	At3g60330
P3A	AtAHA8	948	Q9M2A0	At3g42640
P3A	AtAHA9	954	Q42556	At1g80660
P3A	AtAHA10	947	Q43128	At1g17260
P3A	AtAHA11	956	Q9LV11	At5g62670
P3A	–	813	Q9T0E0^13^	At4g11730
P4	AtALA1	1158	P98204	At5g04930
P4	AtALA2	1107	P98205	At5g44240
P4	AtALA3	1213	Q9XIE6	At1g59820
P4	AtALA4	1216	Q9LNQ4	At1g17500
P4	AtALA5	1228	Q9SGG3	At1g72700
P4	AtALA6	1240	Q9SLK6	At1g54280
P4	AtALA7	1247	Q9LVK9	At3g13900
P4	AtALA8	1189	Q9LK90	At3g27870
P4	AtALA9	1200	Q9SX33	At1g68710
P4	AtALA10	1202	Q9LI83	At3g25610
P4	AtALA11	1203	Q9SAF5	At1g13210
P4	AtALA12	1184	P57792	At1g26130
P5A	AtP5A1	1179	Q9LT02	At5g23630

*^1^Fragment*.

*^2^A possible chimera. The last 200 amino acid residues do not match AtAHA4. Similarity ends at the position equivalent to position 862 in AtAHA4*.

*^3^Possibly a fragment. Maybe 80 amino acid residues missing from C-terminus (when compared to AtAHA2). The similarity extends to position 864 in AtAHA2*.

*^4^A possible fragment or pseudogene. When compared to AtHMA8/PAA1, positions 523–827 are missing (this includes the motif DKTGT). The last 60 amino acid residues are also missing*.

*^5^200 amino acid residues from the C-terminal are missing*.

*^6^No corresponding UniProtKB entry*.

*^7^When compared to AtACA9, amino acid residues 1–170 are missing*.

*^8^Fragment with insert. Similar to AtACA10 (107–529 + 576–1046; the positions on *C. reinhardtii* 3–462 + 856–1347). The UniProtKB entry only covers part of the protein taken from Phytozome*.

*^9^Possibly a pseudogene or chimera. Partly covered by A8I6H0 (460–576) and A8I6H2 (1521–1622). Many regions of low complexity. Similar to AtAHA10 (58–209 + 584–974, AtAHA10 numbering)*.

*^10^First 130 aa missing compared to AtACA8*.

*^11^Near identical copies of most *S. moellendorffii* genes are present in databases indicating co-sequencing of two related cultivars*.

*^12^This protein is not likely to function as an ATPase as several key features are missing including core sequence 2 (TGES− > TASD), core seq. 4 (PEGL− > PVGL, core seq. 5 (the entire intron including DKTGTLT is missing), core sequence 6 (TGDN− > TDND), and core sequence 7 (VVAVTGDGTNDAPAL− > IVAATGMGIHDPKTL). cDNA: AY078942 (613–1049, but different splice variant), CB264713 (772–980), this splice variant. Probably similar to PMAX*.

*^13^Probable pseudogene (Axelsen and Palmgren, 2001)*.

Angiosperm plasma membrane H^+^-ATPases are regulated by an extended C-terminal domain that functions as a pump auto inhibitor (Palmgren et al., 1991). All residues in this domain of ∼100 residues have been mutagenized and two clusters of autoinhibitory sequences have been identified, Region I and Region II (Axelsen et al., 1999). Further, in the extreme C-terminal end, a 14-3-3 binding site has been identified. 14-3-3 binding results in pump activation, but in order for 14-3-3 binding to occur, the penultimate residue (a Thr or Ser) first has to become phosphorylated (Fuglsang et al., 1999; Svennelid et al., 1999; Maudoux et al., 2000).

**Figure 11:**
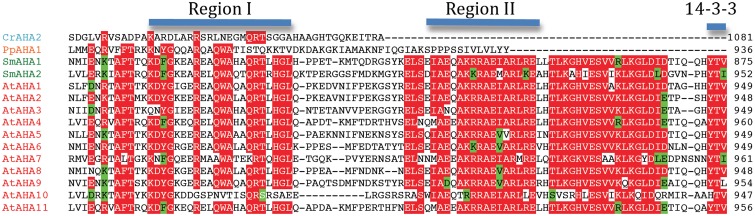
**Alignment of putative autoinhibitory regions in the C-terminal region of P3A H^+^-ATPases**.

When C-terminal sequences of putative P3A ATPases were analyzed, the complete set of regulatory sequences (Region I–II and the 14-3-3 binding site) could be identified in all AHAs of *S. moellendorffii* and *A. thaliana* (Figure 11). In *S. moellendorffii* the shorter C-terminal regions of CrAHA2 and PpAHA1, a stretch of residues with weak but notable similarity to Region I could be identified (Figure 11). In these pumps, sequences with similarity to Region II and the 14-3-3 binding site could not be observed. In Chlorophyte P3A ATPases resembling protist plasma membrane H^+^-ATPases (OtAHA1, CrAHA1, and CrAHA3) no sequences with similarity to any of these regions could be identified. The presence of a putative Region I in the C termini of CrAHA2 and PpAHA1 suggests that the basic regulatory apparatus of the higher plant C-terminus could have been present in the first green plants, but that more complex features (e.g., a 14-3-3 binding site) evolved latter in the evolution of vascular plants. In support of an early origin of Region I, the *S. cerevisiae* plasma membrane H^+^-ATPase Pma1p has a short autoinhibitory sequence in its C-terminal domain with weak similarity to Region I, which appears to be involved in regulation of pump efficiency (Portillo et al., 1989; Venema and Palmgren, 1995).

### P4 ATPases: Putative lipid pumps

P4 ATPases in plants have been implicated in flipping phospholipids across biological membranes (Poulsen et al., 2008a). They are completely absent from eubacteria and archaebacteria, whereas in eukaryotes they are typically encoded for by multigene families (Axelsen and Palmgren, 1998).

**Figure 12:**
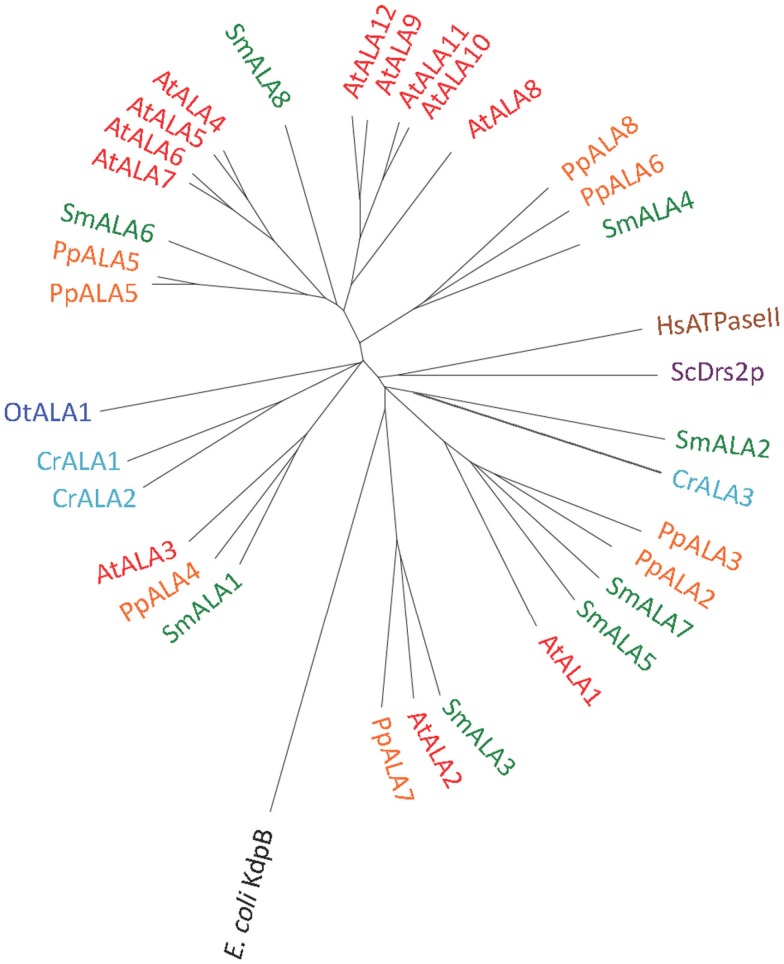
**Phylogenetic tree of P4 ATPases (lipid flippases)**. Accession numbers for sequences are given in Table 2. For comparison, the following outliers were included: A bacterial P1A ATPase *E. coli* KdpB (P03960), the *S. cerevisiae* pump ScDrs2p (P39524), and the *H. sapiens* pump HsATPaseII (ATP8A1; Q9Y2Q0).

We identified P4 ATPases in all of the Viridiplantae investigated in this study (Figure 12). The chlorophyte *O. tauri* was the only organism with a single P4 ATPase. This solitary P4 ATPase groups in the phylogenetic tree in the same larger branch as AtALA3. AtALA3 activity is connected with transport of phosphatidylethanolamine, phosphatidylserine, and phosphatidylcholine in *A. thaliana* (Poulsen et al., 2008b). Further, it is a resident of the trans Golgi of root tip cells where it is connected to the generation of secretory vesicles leaving the Golgi apparatus (Poulsen et al., 2008b).

### P5 ATPases: Pumps with no assigned specificity

P5 ATPases constitute the least characterized group of P-type pumps and their transported ligand – if any – has not been identified. These pumps are absent from prokaryotes and are confined to eukaryotes where they reside in internal membrane systems (Møller et al., 2008). Based on sequence analysis, they are divided into two groups, P5A and P5B, each of which is predicted to transport different substrates based on differences in their transmembrane segments (Figure 14; Sørensen et al., 2010).

**Figure 13:**
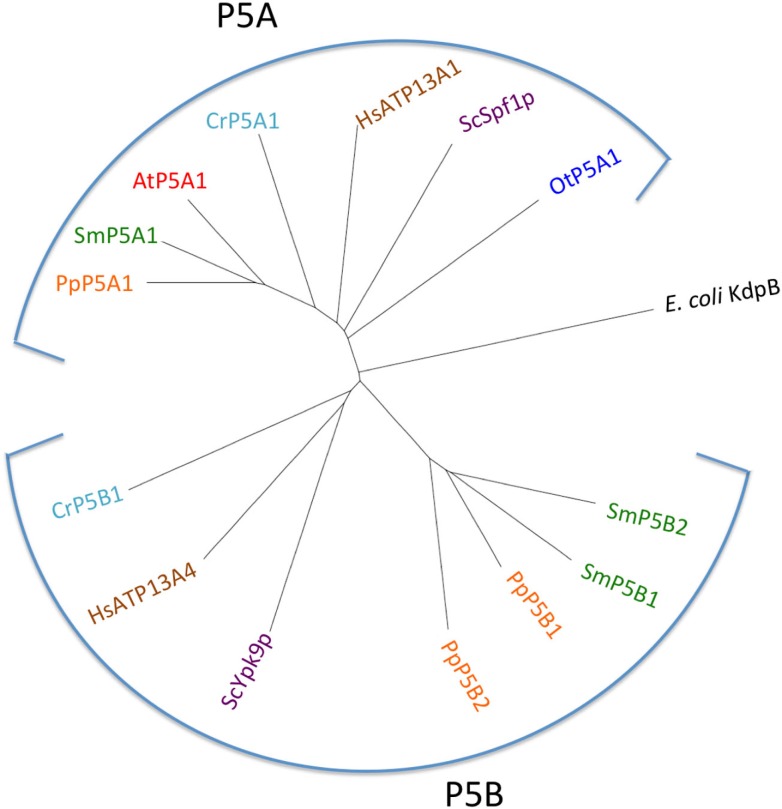
**Phylogenetic tree of P5 ATPases (having unknown transport activity)**. Accession numbers for sequences are given in Table 2. For comparison, the following outliers were included: a bacterial P1A ATPase *E. coli* KdpB (P03960), the *S. cerevisiae* pumps ScSpf1p (P39986) and ScYpk9 (Q12697), and the *H. sapiens* pumps HsATP13A1 (Q6NT90) and HsATP13A4 (Q4VNC1).

P5A ATPases have been found in all eukaryotic genomes analyzed so far (Møller et al., 2008) and were identified in all Viridiplantae analyzed in this study (Figure 13). Only a single P5A ATPase could be identified in each organism.

**Figure 14:**
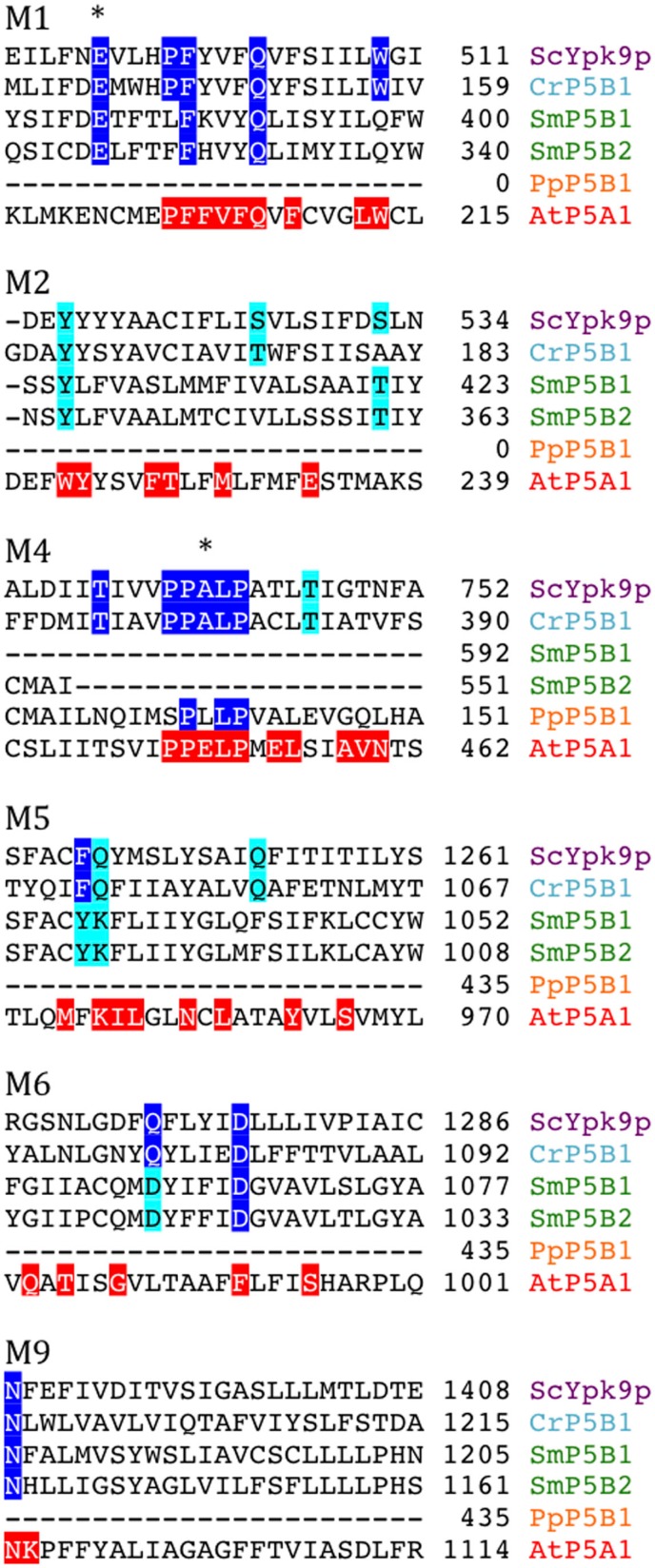
**Alignment of predicted transmembrane segments of putative P5B ATPases aligned with similar regions in the *S. cerevisiae* P5B ATPase ScYpk9p (Q12697)**. Residues conserved in all P5B ATPases (according to Sørensen et al., 2010) are marked in blue. Those that are highly conserved are marked in cyan. The P5A ATPase AtP5A/AtMIA is shown with residues conserved in P5A ATPases highlighted in red (Sørensen et al., 2010). Asterisks mark residues that are likely to play a role in ligand coordination.

P5B ATPase sequences have so far been identified in the genomes of all eukaryotes examined, except for two plant lineages. Their widespread distribution supports a model in which they arose at an early point in the evolution of eukaryotes (Sørensen et al., 2010). When the Viridiplantae genomes analyzed here were searched for P5B sequences, we could identify P5B ATPases in *C. reinhardtii*, *P. patens*, and *S. moellendorffii*, but not in *O. tauri* and *A. thaliana* (Figure 13). This suggests that loss of P5B ATPases occurred at least twice in the evolution of Viridiplantae.

## Discussion

### Evolution of plant P-type ATPases

**Figure 15:**
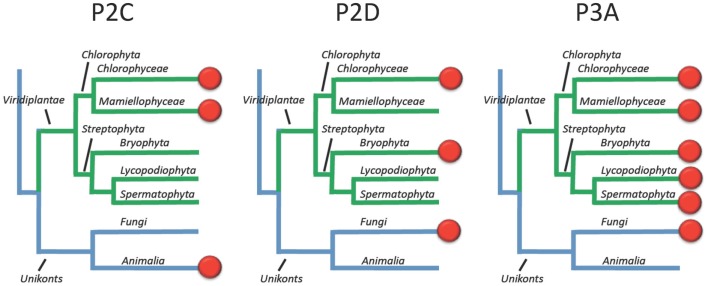
**Overview of the evolution of Na^+^ and H^+^ transporting P-type ATPases in Viridiplantae**. Most likely, the ancestor of green plants had two types of Na^+^ pumps (P2C and P2D) in addition to a plasma membrane H^+^ pump (P3A). In present day plants, the terrestrial green algae *C. reinhardtii* still has all three types of pumps whereas Na^+^ pumps have been lost completely in vascular plants (here represented by Lycopodiophyta and Spermatophyta).

This survey of P-type ATPases provides evidence that members of the green plant lineage require at least five different types of P-type ATPases. Each of the five reference genomes analyzed showed at least one representative from each of the five subgroups of P-type ATPases. However, within the five different subgroups there is still evidence for considerable evolution of biochemical functions (Figure 15). For example, within the P2-type pumps, there are at least four subdivisions that delineate two subfamilies each of Na^+^ and Ca^2+^ pumps (Figure 5).

In *Arabidopsis* there are 46 pumps, compared to 14 in *O. tauri*, 22 in *C. reinhardtii*, 33 in *P. patens*, and 32 in *S. moellendorffii*. This is consistent with a speculation that multicellular organisms require more pumps, presumably to provide specialized functions associated with more complex developmental programs or more variable environments. While loss of function phenotypes have been established for at least one member of each pump type in *Arabidopsis*, most of the 46 pumps remain uncharacterized at the genetic level.

The evolutionary diversity of P-type ATPases raises many questions to be explored. For example, why does *O. tauri* require only one lipid flippase (P4-type pump), whereas rice and *Arabidopsis* have 10 and 12 members, respectively (Baxter et al., 2003). What are the cellular functions of the lipid flippases? Are their biochemical functions limited to flipping lipids, or do they also help insert or remove lipids from membranes? At a structural level, how have the P4-pumps evolved from an ancestor that recognized simple cation substrates into having a dynamic interaction with lipids?

The relative expansion of the proton pump family (P3A) in flowering plants is also noteworthy, with 11 and 10 members in *Arabidopsis* and rice, respectively (Baxter et al., 2003), and only two in *P. patens* and *S. moellendorffii*. Do physiological complexities of flowering plants necessitate different isoform specific features?

In contrast, P5 pumps have only one or two representatives in all five reference organisms. The biochemical functions of these pumps are not clear, but their general importance to eukaryotes is supported by the presence of at least one representative in reference genomes from yeast to man.

The observation that all five reference genomes have two types of Ca^2+^ pumps, P2A and P2B suggests that both subgroups have conserved functions. A role in Ca^2+^ signaling has been proposed for P2B pumps, based on the presence of a regulatory domain that provides for activation of the pump by Ca^2+^/calmodulin. Genetic evidence supports a model in which regulation of pump activity could modulate the magnitude or duration of a Ca^2+^ signal (Qudeimat et al., 2008; Boursiac et al., 2010; Zhu et al., 2010; Spalding and Harper, 2011). For the P2A pumps, genetic evidence suggests that they function in the homeostasis of both Ca^2+^ and Mn^2+^ (Wu et al., 2002; Li et al., 2008; Mills et al., 2008). Delineating signaling and nutritional functions of these pumps is an important challenge for the future.

### The evolution of plasma membrane H^+^ and Na^+^ pumps

The evolution of Na^+^ pumps (type 2C and 2D) is of interest from several perspectives. First, the relatively close relationship between Na^+^ and Ca^2+^ pumps raises an interesting evolutionary question of which came first? Since both Na^+^ and Ca^2+^ can be toxic within the cytosol, did both types of pumps arise early in evolution as a way to efflux toxic ions? Second, it appears that ancestral plants had both P2C and P2D ATPases. Both groups of pumps have remained in Chlorophyceae, represented here by the terrestrial green algae *C. reinhardtii*, but appear to have been lost in vascular plants (Figure 15).

An interesting question is why do the chlorophytes examined here have both H^+^ and Na^+^ pumps? For organisms such a *C. reinhardtii* that can live in fresh water, what is the evolutionary pressure for the retention of a Na^+^ pump? For organisms that live in saline environments, such as *O. tauri*, why retain a plasma membrane H^+^ pump? It seems reasonable to assume that Na^+^ pumps alone could control cytoplasmic Na^+^ levels and energize the plasma membrane for signaling and co-transport systems, as they do in typical animal cells. However, it is possible that the H^+^ pumps actually evolved in marine organisms not to energize the plasma membrane, but rather to control cytoplasmic pH.

Regardless of their evolutionary origins, the observation that both H^+^ and Na^+^ pumps co-exist in the plasma membrane of well studied chlorophytes raises a question about which ion is used to drive secondary active transport systems? Are there different sets of H^+^ or Na^+^ specific co-transporters, or can the cotransporters be driven by either H^+^ or Na^+^ gradients? In vascular plants, Na^+^ pumps appear to have been lost, leaving only plasma membrane H^+^-ATPases to drive secondary transport systems. As a consequence it is thought that most plasma membrane cotransporters in vascular plants are H^+^ coupled.

In the *Arabidopsis* pump AtAHA2, a conserved Arg in transmembrane segment M5 (Arg655) appears to be an important part of the H^+^ pumping apparatus (Pedersen et al., 2007). This residue has been proposed to serve as a built-in cation that allows for rapid transition of the pump from the E1P to the E2P conformational state and as a gate-keeper that prevents H^+^ from flowing backward (“backflow protection”; Pedersen et al., 2007; Buch-Pedersen et al., 2009). Further, in AHA2, a complex C-terminal regulatory domain with two autoinhibitory regions and a binding site for activating 14-3-3 protein is present (Axelsen et al., 1999). Backflow protection and multiple regulatory features are characteristic of all well studied H^+^ pumps in flowering plants. The observation of similar features associated with the *S. moellendorffi* pumps (Figure 11), suggests that H^+^ pumps with “advanced features” may be universal to all vascular plants.

In contrast, the plasma membrane H^+^ pumps in chlorophytes appear to be more primitive in at least two aspects (Figure 9B). First, both P3A pumps of *O. taurii* lack the conserved Arg of M5 and two out of the three P3A pumps in *C. reinhardtii* also lack this residue (Figure 10). Second, a complex C-terminal regulatory domain is absent from the chlorophyte pumps.

The presence of “more advanced” H^+^ pumps in vascular plants would suggest that these organisms have the capacity to utilize H^+^ pumps to energize their plasma membranes with large electrochemical gradients. In flowering plants, H^+^ pumps can create steep H^+^ gradients and membrane potentials that can exceed 200 mV (negative on the inside; Hirsch et al., 1998). In comparison, Na^+^/K^+^ pumps in animal cells typically only produce membrane potentials around 60 mV. It is tempting to hypothesize that vascular plants have evolved to rely on these “more advanced” pumps to create very large electrochemical gradients for special purposes, such as signaling or nutrient transport. However, during evolution, large H^+^-based electrochemical gradients may have been problematic in the presence of a Na^+^ pump. For example, a large membrane potential or pH gradient may have resulted in ancestral Na^+^ pumps mis-functioning as H^+^ or Na^+^ leaks. As a result, Na^+^ pumps may have been lost as vascular plants evolved to rely on their more advanced H^+^ pumps.

### Frontiers in P-type ATPase research

The frontiers of P-type ATPase research can be divided into three areas. The first is to understand cellular and organismal functions of each pump. The second is to match biological functions with a structural understanding of how pumps transport specific substrates, and how their activities are modulated by signaling systems. The third is to explore ideas about how P-type ATPases can be altered or used to improve crop plants. For example, can Na^+^ pumps from chlorophytes be moved into crop plants to provide improved Na^+^ tolerance? Or would these pumps require re-engineering to prevent them from becoming H^+^ or Na^+^ leaks in cell types with large membrane potentials or H^+^ gradients. As these frontiers are considered, it is worth remembering that P-type ATPases have already proven themselves as flexible substrates for evolution, producing pumps with a wide diversity of functions, from pumping protons to flipping lipids.

## Conflict of Interest Statement

The authors declare that the research was conducted in the absence of any commercial or financial relationships that could be construed as a potential conflict of interest.

## Supplementary Material

The Supplementary Material for this article can be found online at http://www.frontiersin.org/Plant_Physiology/10.3389/fpls.2012.00031/abstract

## Footnotes

^1^http://www.phytozome.net/

^2^http://genome.jgipsf.org/Selmo1/Selmo1.home.html

## References

[B1] Abdel-Ghany S. E. Muller-Moule P. Niyogi K. K. Pilon M. Shikanai T. (2005). Two P-type ATPases are required for copper delivery in *Arabidopsis thaliana* chloroplasts. Plant Cell 17, 1233–1251 10.1105/tpc.104.030452 15772282 PMC1087999

[B2] Antebi A. Fink G. R. (1992). The yeast Ca^2+^-ATPase homologue, PMR1, is required for normal Golgi function and localizes in a novel Golgi-like distribution. Mol. Biol. Cell 3, 633–654 1379856 10.1091/mbc.3.6.633 PMC275619

[B3] Argüello J. M. Eren E. González-Guerrero M. (2007). The structure and function of heavy metal transport P_1B_-ATPases. Biometals 20, 233–248 10.1007/s10534-006-9055-6 17219055

[B4] Axelsen K. B. Palmgren M. G. (1998). Evolution of substrate specificities in the P-Type ATPase superfamily. J. Mol. Evol. 46, 84–101 10.1007/PL00006286 9419228

[B5] Axelsen K. B. Palmgren M. G. (2001). Inventory of the superfamily of P-type ion pumps in *Arabidopsis*. Plant Physiol. 126, 696–706 10.1104/pp.126.2.696 11402198 PMC111160

[B6] Axelsen K. B. Venema K. Jahn T. Baunsgaard L. Palmgren M. G. (1999). Molecular dissection of the C-terminal regulatory domain of the plant plasma membrane H^+^-ATPase AHA2: mapping of residues that when altered give rise to an activated enzyme. Biochemistry 38, 7227–7234 10.1021/bi982482l 10353834

[B7] Bækgaard L. Luoni L. De Michelis M. I. Palmgren M. G. (2006). The plant plasma membrane Ca^2+^ pump ACA8 contains overlapping as well as physically separated autoinhibitory and calmodulin-binding domains. J. Biol. Chem. 281, 1058–1065 10.1074/jbc.M508299200 16267044

[B8] Banks J. A. (2009). *Selaginella* and 400 million years of separation. Annu. Rev. Plant Biol. 60, 223–238 10.1146/annurev.arplant.59.032607.092851 19575581

[B9] Barrero-Gil J. Garciadeblás B. Benito B. (2005). Sodium, potassium-ATPases in algae and oomycetes. J. Bioenerg. Biomembr. 37, 269–278 10.1007/s10863-005-6637-x 16167182

[B10] Baxter I. Tchieu J. Sussman M. R. Boutry M. Palmgren M. G. Gribskov M. Harper J. F. Axelsen K. B. (2003). Genomic comparison of P-type ATPase ion pumps in *Arabidopsis* and rice. Plant Physiol. 132, 618–628 10.1104/pp.103.021923 12805592 PMC167002

[B11] Boursiac Y. Lee S. M. Romanowsky S. Blank R. Sladek C. Chung W. S. Harper J. F. (2010). Disruption of the vacuolar calcium-ATPases in *Arabidopsis* results in the activation of a salicylic acid-dependent programmed cell death pathway. Plant Physiol. 154, 1158–1171 10.1104/pp.110.159038 20837703 PMC2971596

[B12] Buch-Pedersen M. J. Pedersen B. P. Veierskov B. Nissen P. Palmgren M. G. (2009). Protons and how they are transported by proton pumps. Pflugers Arch. 457, 573–579 10.1007/s00424-008-0503-8 18458946

[B13] Campbell A. M. Coble A. J. Cohen L. D. Ch’ng T. H. Russo K. M. Long E. M. Armburst E. V. (2001). Identification and DNA sequence of a new H^+^-ATPase in the unicellular green alga *Chlamydomonas reinhardtii* (Chlorophyceae) J. Phycol. 37, 536–542 10.1046/j.1529-8817.2001.037004536.x

[B14] Courties C. Perasso R. Chrétiennot-Dinet M.-J. Gouy M. Guillou L. Troussellier M. (1998). Phylogenetic analysis and genome size of *Ostreococcus tauri* (Chlorophyta, Prasinophyceae). J. Phycol. 34, 844–849 10.1046/j.1529-8817.1998.340844.x

[B15] Curran A. C. Hwang I. Corbin J. Martinez S. Rayle D. Sze H. Harper J. F. (2000). Autoinhibition of a calmodulin-dependent calcium pump involves a structure in the stalk that connects the transmembrane domain to the ATPase catalytic domain. J. Biol. Chem. 275, 30301–30308 10.1074/jbc.M002047200 10818096

[B16] Derelle E. Ferraz C. Rombauts S. Rouzé P. Worden A. Z. Robbens S. Partensky F. Degroeve S. Echeynié S. Cooke R. Saeys Y. Wuyts J. Jabbari K. Bowler C. Panaud O. Piégu B. Ball S. G. Ral J. P. Bouget F. Y. Piganeau G. De Baets B. Picard A. Delseny M. Demaille J. Van de Peer Y. Moreau H. (2006). Genome analysis of the smallest free-living eukaryote *Ostreococcus tauri* unveils many unique features. Proc. Natl. Acad. Sci. U.S.A. 103, 11647–11652 10.1073/pnas.0604795103 16868079 PMC1544224

[B17] Dutta S. J. Liu J. Hou Z. Mitra B. (2006). Conserved aspartic acid 714 in transmembrane segment 8 of the ZntA subgroup of P_1B_-type ATPases is a metal-binding residue. Biochemistry 45, 5923–5931 10.1021/bi0523456 16669635

[B18] Edgar R. C. (2004). MUSCLE: multiple sequence alignment with high accuracy and high throughput. Nucleic Acids Res. 32, 1792–1797 10.1093/nar/gkh180 15034147 PMC390337

[B19] Flowers T. J. Colmer T. D. (2008). Salinity tolerance in halophytes. New Phytol. 179, 945–963 10.1111/j.1469-8137.2008.02531.x 18565144

[B20] Fuglsang A. T. Visconti S. Drumm K. Jahn T. Stensballe A. Mattei B. Jensen O. N. Aducci P. Palmgren M. G. (1999). Binding of 14-3-3 protein to the plasma membrane H^+^-ATPase AHA2 involves the three C-terminal residues Tyr^946^-Thr-Val and requires phosphorylation of Thr947. J. Biol. Chem. 274, 36774–36780 10.1074/jbc.274.51.36774 10593986

[B21] Gimmler H. (2000). Primary sodium plasma membrane ATPases in salt-tolerant algae: facts and fictions. J. Exp. Bot. 51, 1171–1178 10.1093/jexbot/51.348.1171 10937692

[B22] Gonzalez R. J. (2011). The physiology of hyper-salinity tolerance in teleost fish: a review. J. Comp. Physiol. B Biochem. Syst. Environ. Physiol. (in press). 10.1007/s00360-011-0624-9 22033744

[B23] Harper J. F. Hong B. Hwang I. Guo H. Q. Stoddard R. Huang J. F. Palmgren M. G. Sze H. (1998). A novel calmodulin-regulated Ca^2+^-ATPase (ACA2) from *Arabidopsis* with an N-terminal autoinhibitory domain. J. Biol. Chem. 273, 1099–1106 10.1074/jbc.273.2.1099 9422775

[B24] Haruta M. Burch H. L. Nelson R. B. Barrett-Wilt G. Kline K. G. Mohsin S. B. Young J. C. Otegui M. S. Sussman M. R. (2010). Molecular characterization of mutant *Arabidopsis* plants with reduced plasma membrane proton pump activity. J. Biol. Chem. 285, 17918–17929 10.1074/jbc.M110.101733 20348108 PMC2878554

[B25] Hirsch R. E. Lewis B. D. Spalding E. P. Sussman M. R. (1998). A role for the AKT1 potassium channel in plant nutrition. Science 280, 918–921 10.1126/science.280.5365.918 9572739

[B26] Howe K. Bateman A. Durbin R. (2002). QuickTree: building huge neighbour-joining trees of protein sequences. Bioinformatics 18, 1546–1547 10.1093/bioinformatics/18.11.1546 12424131

[B27] Huson D. H. Richter D. C. Rausch C. Dezulian T. Franz M. Rupp R. (2007). Dendroscope: an interactive viewer for large phylogenetic trees. BMC Bioinformatics 8, 460 10.1186/1471-2105-8-460 18034891 PMC2216043

[B28] Hussain D. Haydon M. J. Wang Y. Wong E. Sherson S. M. Young J. Camakaris J. Harper J. F. Cobbett C. S. (2004). P-type ATPase heavy metal transporters with roles in essential zinc homeostasis in *Arabidopsis*. Plant Cell 16, 1327–1339 10.1105/tpc.020487 15100400 PMC423219

[B29] Hwang I. Harper J. F. Liang F. Sze H. (2000). Calmodulin activation of an endoplasmic reticulum-located calcium pump involves an interaction with the N-terminal autoinhibitory domain. Plant Physiol. 122, 157–168 10.1104/pp.122.1.157 10631259 PMC58854

[B30] James P. Maeda M. Fischer R. Verma A. Krebs J. Penniston J. Carafoli E. (1988). Identification and primary structure of a calmodulin binding domain of the Ca^2+^ pump of human erythrocytes. J. Biol. Chem. 263, 2905–2910 2963820

[B31] Kim Y. Y. Choi H. Segami S. Cho H. T. Martinoia E. Maeshima M. Lee Y. (2009). AtHMA1 contributes to the detoxification of excess Zn(II) in *Arabidopsis*. Plant J. 58, 737–753 10.1111/j.1365-313X.2008.03770.x 19207208

[B32] Li X. Chanroj S. Wu Z. Romanowsky S. M. Harper J. F. Sze H. (2008). A distinct endosomal Ca^2+^/Mn^2+^ pump affects root growth through the secretory process. Plant Physiol. 147, 1675–1689 10.1104/pp.108.119909 18567829 PMC2492598

[B33] Luo S. Fang J. Docampo R. (2006). Molecular characterization of *Trypanosoma brucei* P-type H^+^-ATPases. J. Biol. Chem. 281, 21963–21973 10.1074/jbc.M601559200 16757482

[B34] Luo S. Scott D. A. Docampo R. (2002). Trypanosoma cruzi H^+^-ATPase 1 (TcHA1) and 2 (TcHA2) genes complement yeast mutants defective in H^+^ pumps and encode plasma membrane P-type H^+^-ATPases with different enzymatic properties. J. Biol. Chem. 277, 44497–44506 10.1074/jbc.M111072200 12221074

[B35] Malmström S. Askerlund P. Palmgren M. G. (1997). A calmodulin-stimulated Ca^2+^-ATPase from plant vacuolar membranes with a putative regulatory domain at its N-terminus. FEBS Lett. 400, 324–328 10.1016/S0014-5793(96)01448-2 9009223

[B36] Maudoux O. Batoko H. Oecking C. Gevaert K. Vandekerckhove J. Boutry M. Morsomme P. (2000). A plant plasma membrane H^+^-ATPase expressed in yeast is activated by phosphorylation at its penultimate residue and binding of 14-3-3 regulatory proteins in the absence of fusicoccin. J. Biol. Chem. 275, 17762–17770 10.1074/jbc.M909690199 10748153

[B37] Merchant S. S. Prochnik S. E. Vallon O. Harris E. H. Karpowicz S. J. Witman G. B. Terry A. Salamov A. Fritz-Laylin L. K. Maréchal-Drouard L. Marshall W. F. Qu L. H. Nelson D. R. Sanderfoot A. A. Spalding M. H. Kapitonov V. V. Ren Q. Ferris P. Lindquist E. Shapiro H. Lucas S. M. Grimwood J. Schmutz J. Cardol P. Cerutti H. Chanfreau G. Chen C. L. Cognat V. Croft M. T. Dent R. Dutcher S. Fernández E. Fukuzawa H. González-Ballester D. González-Halphen D. Hallmann A. Hanikenne M. Hippler M. Inwood W. Jabbari K. Kalanon M. Kuras R. Lefebvre P. A. Lemaire S. D. Lobanov A. V. Lohr M. Manuell A. Meier I. Mets L. Mittag M. Mittelmeier T. Moroney J. V. Moseley J. Napoli C. Nedelcu A. M. Niyogi K. Novoselov S. V. Paulsen I. T. Pazour G. Purton S. Ral J. P. Riaño-Pachón D. M. Riekhof W. Rymarquis L. Schroda M. Stern D. Umen J. Willows R. Wilson N. Zimmer S. L. Allmer J. Balk J. Bisova K. Chen C. J. Elias M. Gendler K. Hauser C. Lamb M. R. Ledford H. Long J. C. Minagawa J. Page M. D. Pan J. Pootakham W. Roje S. Rose A. Stahlberg E. Terauchi A. M. Yang P. Ball S. Bowler C. Dieckmann C. L. Gladyshev V. N. Green P. Jorgensen R. Mayfield S. Mueller-Roeber B. Rajamani S. Sayre R. T. Brokstein P. Dubchak I. Goodstein D. Hornick L. Huang Y. W. Jhaveri J. Luo Y. Martínez D. Ngau W. C. Otillar B. Poliakov A. Porter A. Szajkowski L. Werner G. Zhou K. Grigoriev I. V. Rokhsar D. S. Grossman A. R. (2007). The *Chlamydomonas* genome reveals the evolution of key animal and plant functions. Science 318, 245–250 10.1126/science.1143609 17932292 PMC2875087

[B38] Mills R. F. Doherty M. L. López-Marqués R. L. Weimar T. Dupree P. Palmgren M. G. Pittman J. K. Williams L. E. (2008). ECA3, a Golgi-localized P_2A_-type ATPase, plays a crucial role in manganese nutrition in *Arabidopsis*. Plant Physiol. 146, 116–128 10.1104/pp.107.110817 18024560 PMC2230566

[B39] Mills R. F. Krijger G. C. Baccarini P. J. Hall J. L. Williams L. E. (2003). Functional expression of AtHMA4, a P_1B_-type ATPase of the Zn/Co/Cd/Pb subclass. Plant J. 35, 164–176 10.1046/j.1365-313X.2003.01790.x 12848823

[B40] Møller A. B. Asp T. Holm P. B. Palmgren M. G. (2008). Phylogenetic analysis of P_5_ P-type ATPases, a eukaryotic lineage of secretory pathway pumps. Mol. Phylogenet. Evol. 46, 619–634 10.1016/j.ympev.2007.10.023 18155930

[B41] Møller J. V. Juul B. le Maire M. (1996). Structural organization, ion transport, and energy transduction of P-type ATPases. Biochim. Biophys. Acta 1286, 1–51 8634322 10.1016/0304-4157(95)00017-8

[B42] Moreno I. Norambuena L. Maturana D. Toro M. Vergara C. Orellana A. Zurita-Silva A. Ordenes V. R. (2008). AtHMA1 is a thapsigargin-sensitive Ca^2+^/heavy metal pump. J. Biol. Chem. 283, 9633–9641 10.1074/jbc.M800736200 18252706

[B43] Morth J. P. Pedersen B. P. Buch-Pedersen M. J. Andersen J. P. Vilsen B. Palmgren M. G. Nissen P. (2011). A structural overview of the plasma membrane Na^+^,K^+^-ATPase and H^+^-ATPase ion pumps. Nat. Rev. Mol. Cell Biol. 12, 60–70 10.1038/nrm3031 21179061

[B44] Morth J. P. Pedersen B. P. Toustrup-Jensen M. S. Sørensen T. L. Petersen J. Andersen J. P. Vilsen B. Nissen P. (2007). Crystal structure of the sodium-potassium pump. Nature 450, 1043–1049 10.1038/nature06419 18075585

[B45] Olesen C. Picard M. Winther A. M. Gyrup C. Morth J. P. Oxvig C. Møller J. V. Nissen P. (2007). The structural basis of calcium transport by the calcium pump. Nature 450, 1036–1042 10.1038/nature06418 18075584

[B46] Palmgren M. G. (2001). Plant plasma membrane H^+^-ATPases: powerhouses for nutrient uptake. Annu. Rev. Plant Physiol. Plant Mol. Biol. 52, 817–845 10.1146/annurev.arplant.52.1.817 11337417

[B47] Palmgren M. G. Harper J. F. (1999). Pumping with plant P-type ATPases. J. Exp. Bot. 50, 883–893 10.1093/jexbot/50.suppl_1.883

[B48] Palmgren M. G. Sommarin M. Serrano R. Larsson C. (1991). Identification of an autoinhibitory domain in the C-terminal region of the plant plasma membrane H^+^-ATPase. J. Biol. Chem. 266, 20470–20475 1834646

[B49] Pedersen B. P. Buch-Pedersen M. J. Morth J. P. Palmgren M. G. Nissen P. (2007). Crystal structure of the plasma membrane proton pump. Nature 450, 1111–1114 10.1038/nature06417 18075595

[B50] Popova L. Balnokin Y. Dietz K. J. Gimmler H. (1999). Characterization of phosphorylated intermediates synthetized during the catalytic cycle of the sodium adenosine triphosphatase in the plasma membrane of the marine unicellular alga *Tetraselmis (Platymonas) viridis*. J. Plant Physiol. 155, 302–309 10.1016/S0176-1617(99)80109-6

[B51] Popova L. G. Shumkova G. A. Andreev I. M. Balnokin Y. V. (2005). Functional identification of electrogenic Na^+^-translocating ATPase in the plasma membrane of the halotolerant microalga *Dunaliella maritima*. FEBS Lett. 579, 5002–5006 10.1016/j.febslet.2005.07.087 16137688

[B52] Portillo F. de Larrinoa I. F. Serrano R. (1989). Deletion analysis of yeast plasma membrane H^+^-ATPase and identification of a regulatory domain at the carboxyl-terminus. FEBS Lett. 247, 381–385 10.1016/0014-5793(89)81375-4 2523820

[B53] Poulsen L. R. López-Marqués R. L. Palmgren M. G. (2008a). Flippases: still more questions than answers. Cell. Mol. Life Sci. 65, 3119–3125 10.1007/s00018-008-8341-6 18791845 PMC11131903

[B54] Poulsen L. R. López-Marqués R. L. McDowell S. C. Okkeri J. Licht D. Schulz A. Pomorski T. Harper J. F. Palmgren M. G. (2008b). The Arabidopsis P4-ATPase ALA3 localizes to the Golgi and requires a β-subunit to function in lipid translocation and secretory vesicle formation. Plant Cell 20, 658–676 10.1105/tpc.107.054767 18344284 PMC2329932

[B55] Qudeimat E. Faltusz A. M. Wheeler G. Lang D. Brownlee C. Reski R. Frank W. (2008). A P_IIB_-type Ca^2+^-ATPase is essential for stress adaptation in *Physcomitrella patens*. Proc. Natl. Acad. Sci. U.S.A. 105, 19555–19560 10.1073/pnas.0800864105 19050080 PMC2614799

[B56] Rensing S. A. Lang D. Zimmer A. D. Terry A. Salamov A. Shapiro H. Nishiyama T. Perroud P. F. Lindquist E. A. Kamisugi Y. Tanahashi T. Sakakibara K. Fujita T. Oishi K. Shin-I T. Kuroki Y. Toyoda A. Suzuki Y. Hashimoto S. Yamaguchi K. Sugano S. Kohara Y. Fujiyama A. Anterola A. Aoki S. Ashton N. Barbazuk W. B. Barker E. Bennetzen J. L. Blankenship R. Cho S. H. Dutcher S. K. Estelle M. Fawcett J. A. Gundlach H. Hanada K. Heyl A. Hicks K. A. Hughes J. Lohr M. Mayer K. Melkozernov A. Murata T. Nelson D. R. Pils B. Prigge M. Reiss B. Renner T. Rombauts S. Rushton P. J. Sanderfoot A. Schween G. Shiu S. H. Stueber K. Theodoulou F. L. Tu H. Van de Peer Y. Verrier P. J. Waters E. Wood A. Yang L. Cove D. Cuming A. C. Hasebe M. Lucas S. Mishler B. D. Reski R. Grigoriev I. V. Quatrano R. S. Boore J. L. (2008). The *Physcomitrella* genome reveals evolutionary insights into the conquest of land by plants. Science 319, 64–69 10.1126/science.1150646 18079367

[B57] Rochaix J. D. (1995). *Chlamydomonas reinhardtii* as the photosynthetic yeast. Annu. Rev. Genet. 29, 209–230 10.1146/annurev.ge.29.120195.001233 8825474

[B58] Rodríguez-Navarro A. Benito B. (2010). Sodium or potassium efflux ATPase – a fungal, bryophyte, and protozoal ATPase. Biochim. Biophys. Acta 1798, 1841–1853 10.1016/j.bbamem.2010.07.009 20650263

[B59] Rodríguez-Navarro A. (2000). Potassium transport in fungi and plants. Biochim. Biophys. Acta 1469, 1–30 10692635 10.1016/s0304-4157(99)00013-1

[B60] Rudolph H. K. Antebi A. Fink G. R. Buckley C. M. Dorman T. E. LeVitre J. Davidow L. S. Mao J. I. Moir D. T. (1989). The yeast secretory pathway is perturbed by mutations in PMR1, a member of a Ca^2+^ ATPase family. Cell 58, 133–145 10.1016/0092-8674(89)90410-8 2526682

[B61] Sáez A. G. Lozano E. Zaldívar-Riverón A. (2009). Evolutionary history of Na,K-ATPases and their osmoregulatory role. Genetica 136, 479–490 10.1007/s10709-009-9356-0 19214758

[B62] Seigneurin-Berny D. Gravot A. Auroy P. Mazard C. Kraut A. Finazzi G. Grunwald D. Rappaport F. Vavasseur A. Joyard J. Richaud P. Rolland N. (2006). HMA1, a new Cu-ATPase of the chloroplast envelope, is essential for growth under adverse light conditions. J. Biol. Chem. 281, 2882–2892 10.1074/jbc.M508333200 16282320

[B63] Serrano R. (1989). Structure and function of proton translocating ATPase in plasma membranes of plants and fungi. Biochim. Biophys. Acta 947, 1–28 2894226 10.1016/0304-4157(88)90017-2

[B64] Skou J. C. (1957). The influence of some cations on an adenosine triphosphatase from peripheral nerves. Biochim. Biophys. Acta 23, 394–401 10.1016/0006-3002(57)90343-8 13412736

[B65] Skulachev V. P. (1988). Membrane Bioenergetics. Berlin: Springer-Verlag

[B66] Sørensen D. M. Buch-Pedersen M. J. Palmgren M. G. (2010). Structural divergence between the two subgroups of P5 ATPases. Biochim. Biophys. Acta 1797, 846–855 10.1016/j.bbabio.2010.04.010 20416272

[B67] Spalding E. P. Harper J. F. (2011). The ins and outs of cellular Ca^2+^ transport. Curr. Opin. Plant Biol. 14, 715–720 10.1016/j.pbi.2011.08.001 21865080 PMC3230696

[B68] Svennelid F. Olsson A. Piotrowski M. Rosenquist M. Ottman C. Larsson C. Oecking C. Sommarin M. (1999). Phosphorylation of Thr-948 at the C terminus of the plasma membrane H^+^-ATPase creates a binding site for the regulatory 14-3-3 protein. Plant Cell 11, 2379–2392 10.1105/tpc.11.12.2379 10590165 PMC144135

[B69] Sze H. Liang F. Hwang I. Curran A. C. Harper J. F. (2000). Diversity and regulation of plant Ca^2+^ pumps: insights from expression in yeast. Annu. Rev. Plant Physiol. Plant Mol. Biol. 51, 433–462 10.1146/annurev.arplant.51.1.433 11543429

[B70] Tester M. Davenport R. (2003). Na^+^ tolerance and Na^+^ transport in higher plants. Ann. Bot. 91, 503–527 10.1093/aob/mcg058 12646496 PMC4242248

[B71] The Arabidopsis Genome Initiative (2000). Analysis of the genome sequence of the flowering plant *Arabidopsis thaliana*. Nature 408, 796–815 10.1038/35048692 11130711

[B72] The UniProt Consortium (2012). Reorganizing the protein space at the Universal Protein Resource (UniProt). Nucleic Acids Res. 40, D71–D75 10.1093/nar/gkr981 22102590 PMC3245120

[B73] Toyoshima C. Nakasako M. Nomura H. Ogawa H. (2000). Crystal structure of the calcium pump of sarcoplasmic reticulum at 2.6 Ǻ resolution. Nature 405, 647–655 10.1038/35015017 10864315

[B74] Venema K. Palmgren M. G. (1995) Metabolic modulation of transport coupling ratio in yeast plasma membrane H^+^-ATPase. J. Biol. Chem. 270, 19659–19667 10.1074/jbc.270.33.19659 7642655

[B75] Verret F. Gravot A. Auroy P. Leonhardt N. David P. Nussaume L. Vavasseur A. Richaud P. (2004). Overexpression of AtHMA4 enhances root-to-shoot translocation of zinc and cadmium and plant metal tolerance. FEBS Lett. 576, 306–312 10.1016/j.febslet.2004.09.023 15498553

[B76] Whittamore J. M. (2012). Osmoregulation and epithelial water transport: lessons from the intestine of marine teleost fish. J. Comp. Physiol. B Biochem. Syst. Environ. Physiol. 182, 1–39 10.1007/s00360-011-0601-3 21735220

[B77] Williams L. E. Mills R. F. (2005). P_1B_-ATPases – an ancient family of transition metal pumps with diverse functions in plants. Trends Plant Sci. 10, 491–502 10.1016/j.tplants.2005.08.008 16154798

[B78] Wong C. K. Cobbett C. S. (2009). HMA P-type ATPases are the major mechanism for root-to-shoot Cd translocation in *Arabidopsis thaliana*. New Phytol. 181, 71–78 10.1111/j.1469-8137.2008.02637.x 19076718

[B79] Wu Z. Liang F. Hong B. Young J. C. Sussman M. R. Harper J. F. Sze H. (2002). An endoplasmic reticulum-bound Ca^2+^/Mn^2+^ pump, ECA1, supports plant growth and confers tolerance to Mn^2+^ stress. Plant Physiol. 130, 128–137 10.1104/pp.004440 12226493 PMC166546

[B80] Wuytack F. Raeymaekers L. Missiaen L. (2003). PMR1/SPCA Ca^2+^ pumps and the role of the Golgi apparatus as a Ca^2+^ store. Pflugers Arch. 446, 148–153 12739151 10.1007/s00424-003-1011-5

[B81] Yamaguchi T. Blumwald E. (2005). Developing salt-tolerant crop plants: challenges and opportunities. Trends Plant Sci. 10, 615–620 10.1016/j.tplants.2005.10.002 16280254

[B82] Yoon H. S. Hackett J. D. Ciniglia C. Pinto G. Bhattacharya D. (2004). A molecular timeline for the origin of photosynthetic eukaryotes. Mol. Biol. Evol. 21, 809–818 10.1093/molbev/msh075 14963099

[B83] Zhu X. Caplan J. Mamillapalli P. Czymmek K. Dinesh-Kumar S. P. (2010). Function of endoplasmic reticulum calcium ATPase in innate immunity-mediated programmed cell death. EMBO J. 29, 1007–1018 10.1038/emboj.2009.402 20075858 PMC2837167

